# AI-assisted prediction of differential response to antidepressant classes using electronic health records

**DOI:** 10.1038/s41746-023-00817-8

**Published:** 2023-04-26

**Authors:** Yi-han Sheu, Colin Magdamo, Matthew Miller, Sudeshna Das, Deborah Blacker, Jordan W. Smoller

**Affiliations:** 1 Center for Precision Psychiatry, Massachusetts General Hospital, Boston, MA USA; 2 Psychiatric and Neurodevelopmental Genetics Unit, Center for Genomic Medicine, Massachusetts General Hospital, Boston, MA USA; 3 Department of Psychiatry, Massachusetts General Hospital / Harvard Medical School, Boston, MA USA; 4 Stanley Center for Psychiatric Research, Broad Institute of MIT and Harvard, Cambridge, MA USA; 5 Department of Neurology, Massachusetts General Hospital / Harvard Medical School, Boston, MA USA; 6 Harvard Injury Control Research Center, Harvard T.H. Chan School of Public Health, Boston, MA USA; 7 Bouvé College of Health Sciences, Northeastern University, Boston, MA USA; 8 Department of Epidemiology, Harvard T.H. Chan School of Public Health, Boston, MA USA

**Keywords:** Medical research, Depression, Prognosis

## Abstract

Antidepressant selection is largely a trial-and-error process. We used electronic health record (EHR) data and artificial intelligence (AI) to predict response to four antidepressants classes (SSRI, SNRI, bupropion, and mirtazapine) 4 to 12 weeks after antidepressant initiation. The final data set comprised 17,556 patients. Predictors were derived from both structured and unstructured EHR data and models accounted for features predictive of treatment selection to minimize confounding by indication. Outcome labels were derived through expert chart review and AI-automated imputation. Regularized generalized linear model (GLM), random forest, gradient boosting machine (GBM), and deep neural network (DNN) models were trained and their performance compared. Predictor importance scores were derived using SHapley Additive exPlanations (SHAP). All models demonstrated similarly good prediction performance (AUROCs ≥ 0.70, AUPRCs ≥ 0.68). The models can estimate differential treatment response probabilities both between patients and between antidepressant classes for the same patient. In addition, patient-specific factors driving response probabilities for each antidepressant class can be generated. We show that antidepressant response can be accurately predicted from real-world EHR data with AI modeling, and our approach could inform further development of clinical decision support systems for more effective treatment selection.

## Introduction

Depression is a common and often disabling psychiatric condition^1^. According to the Centers for Disease Control and Prevention (CDC, USA), more than 10% of adults are prescribed antidepressants within any given 30-day window^2^, making antidepressants one of the most commonly used categories of medications. The American Psychiatric Association guidelines^3^ for treatment of major depressive disorder (MDD) suggests four classes of first-line antidepressant medications: selective serotonin reuptake inhibitors (SSRIs), serotonin-norepinephrine reuptake inhibitors (SNRIs), mirtazapine, and bupropion. Unfortunately, identifying the most effective treatment for a given patient remains a trial-and-error proposition. As antidepressant effectiveness may not be evident before 4–12 weeks, each inadequate trial can incur prolonged morbidity, disability and exposure to adverse effects as well as substantial healthcare costs.

Although average response rates are similar across different antidepressant classes^4^, individual response can vary widely in clinical practice. Currently, clinicians initiating antidepressant treatment have few tools or evidence-based predictors on which to rely. A major goal of precision psychiatry is to optimize treatment matching using patient-specific profiles. The growing availability of large-scale health data such as electronic health records (EHRs) coupled with advances in machine learning offer new opportunities for developing clinical decision support tools that may address this challenge^5–8^.

That said, setting up an accurate and scalable system to guide antidepressant selection poses specific challenges. First, patient characteristics that may contribute to response prediction, such as depression symptomatology, are not readily codified. Second, the gold standard for characterizing antidepressant response from the EHR remains expert chart review, which is labor- and time-intensive. Lastly, in contrast to modeling predictions under the naturalistic, observational setting, modeling for interventional recommendations requires additional consideration of potential confounds that could arise from non-random treatment assignments. While a handful of studies have modeled prediction of antidepressant response using data specifically collected for research such as brain imaging or EEG, sample sizes have been typically modest and these approaches can be costly and difficult to scale^9–42^. Ideally, a clinically useful model would enable accurate prediction of antidepressant response, comparative predicted response for alternative treatment choices, and control for potential confounding – in particular, confounding by indication – that is, pretreatment factors associated with both the propensity to choose an antidepressant and treatment response.

To address these limitations, we developed a machine learning (ML) pipeline to predict antidepressant response using real-world, large-scale EHR data. The pipeline incorporates the following features: (1) a battery of prediction models with different levels of complexity, ranging from linear to highly non-linear models such as deep neural networks^43^ (DNNs, often referred to as “AI”), which enables comparative model performance; (2) an AI-based natural language processing (NLP) proxy labeling system, as a complement to expert label curation, to enable scalable model training; (3) confounding control through explicit inclusion of potential confounders among the predictors; (4) direct incorporation of unstructured data (i.e., clinical notes) as an additional predictor component to maximize use of information contained in EHRs.

We use the ML pipeline to address the following scientific questions: (1) Can clinical data routinely obtained before initial treatment with an antidepressant predict outcomes within 4–12 weeks after initiation? (2) Can such information predict which class of medication would work better for a particular patient? (3) What might be the best model architecture to do so? As most antidepressant prescriptions are initiated in non-psychiatric settings^44^, we focused on patients for whom treatment was started by a non-psychiatrist physician (e.g. primary care physician).

See Supplementary Note 1 for brief description of DNN and the DNN classes we used in this study.

## Results

### Patient characteristics

**Fig. 1:**
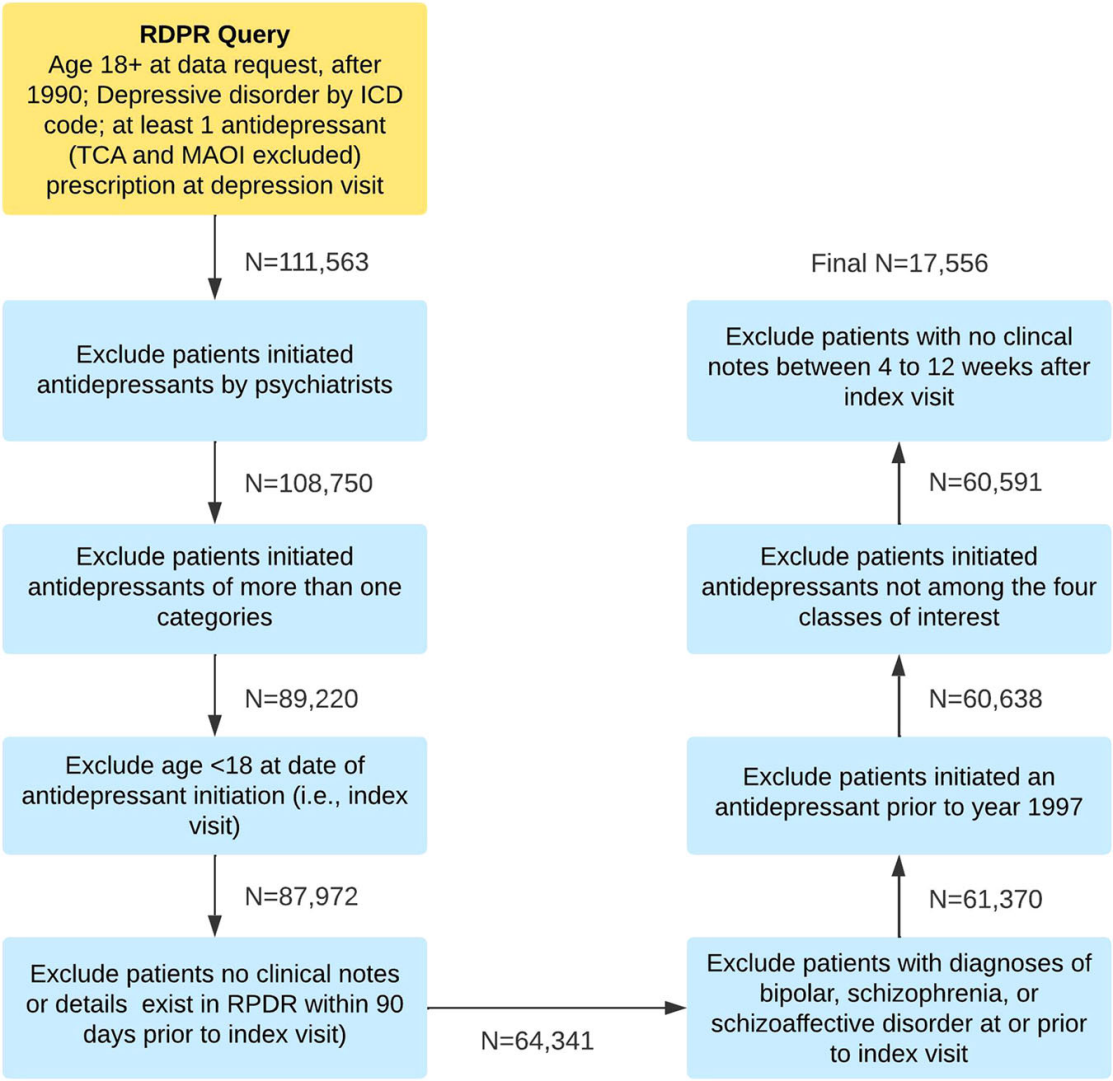
Flowchart for sample selection after sequential application of exclusion criteria. Text boxes denote each step for sampleselection. The numbers next to the arrows represent the number of patients remaining after applying the selection step in the preceding text box.

The EHR data query for the period of 1990–2018 retrieved 111,563 adult patients who had at least one International Classification of Diseases (ICD) code for depression and received a new antidepressant prescription at the same visit. After applying our exclusion criteria, a total of 17,556 patients were included in the analysis (Fig. 1). We annotated 3600 patients (details provided in the Methods section) with expert-curated outcome labels (46% labeled positive) and the remainder with imputed labels (42% labeled positive). Patient characteristics, overall and stratified by the four antidepressant classes, are provided in Table 1.

**Table 1:** Patient characteristics overall and by antidepressant class.

Class of first antidepressant prescribed	*Any*	*Bupropion*	*Mirtazapine*	*SNRI*	*SSRI*
**Antidepressant category first prescribed**	*No.(%)*				
Bupropion	1765 (10.05)				
Mirtazapine	874 (4.98)				
SNRI	1635 (9.31)				
SSRI	13282 (75.66)				
**Demographics**					
*Gender*	*No.(%)*	*No. (%)*	*No. (%)*	*No. (%)*	*No. (%)*
Male	5974 (34.03)	689 (39.04)	405 (46.34)	491 (30.03)	4389 (33.04)
Female	11582 (65.97)	1076 (60.96)	469 (53.66)	1144 (69.97)	8893 (66.96)
*Race*	*No.(%)*	*No.(%)*	*No.(%)*	*No.(%)*	*No.(%)*
African American	1185 (6.75)	111 (6.29)	75 (8.58)	96 (5.87)	903 (6.8)
Asian	374 (2.13)	29 (1.64)	26 (2.97)	19 (1.16)	300 (2.26)
Caucasian	13197 (75.17)	1387 (78.58)	627 (71.74)	1333 (81.53)	9850 (74.16)
Hispanic	1558 (8.87)	125 (7.08)	79 (9.04)	84 (5.14)	1270 (9.56)
Other	781 (4.45)	69 (3.91)	36 (4.12)	69 (4.22)	607 (4.57)
Unknown	461 (2.63)	44 (2.49)	31 (3.55)	34 (2.08)	352 (2.65)
*Marital status*	*No.(%)*	*No.(%)*	*No.(%)*	*No.(%)*	*No.(%)*
Single	6038 (34.39)	664 (37.62)	257 (29.41)	578 (35.35)	4539 (34.17)
Married/Partner	7519 (42.83)	734 (41.59)	346 (39.59)	697 (42.63)	5742 (43.23)
Other	108 (0.62)	15 (0.85)	2 (0.23)	10 (0.61)	81 (0.61)
Separated/Divorced	2070 (11.79)	208 (11.78)	107 (12.24)	234 (14.31)	1521 (11.45)
Unknown	333 (1.9)	40 (2.27)	24 (2.75)	27 (1.65)	242 (1.82)
Widowed	1488 (8.48)	104 (5.89)	138 (15.79)	89 (5.44)	1157 (8.71)
*Primary language*	*No.(%)*	*No.(%)*	*No.(%)*	*No.(%)*	*No.(%)*
English	15265 (86.95)	1602 (90.76)	740 (84.67)	1488 (91.01)	11435 (86.09)
Other	1914 (10.9)	128 (7.25)	110 (12.59)	110 (6.73)	1566 (11.79)
Unknown	377 (2.15)	35 (1.98)	24 (2.75)	37 (2.26)	281 (2.12)
**Antidepressant and other prescriptions**	Mean(SD)	Mean(SD)	Mean(SD)	Mean(SD)	Mean(SD)
Age at antidepressant initiation	50.04 (17.59)	47.19 (15.99)	60.33 (18.95)	49.63 (15.79)	49.79 (17.68)
Number of co-occurring medications	21.64 (21.12)	19.48 (18.85)	32.8 (27.67)	23.67 (20.76)	20.94 (20.71)
Number of NSAIDs	0.79 (1.66)	0.83 (1.68)	0.93 (2.1)	0.99 (1.98)	0.75 (1.58)
**Depression related symptoms**^**a**^					
Depressive mood symptoms (0-7)	0.95 (1.21)	0.86 (1.19)	1.24 (1.36)	0.82 (1.16)	0.95 (1.2)
Anxiety symptoms (0-1)	0.76 (0.43)	0.74 (0.44)	0.81 (0.4)	0.77 (0.42)	0.75 (0.43)
Pain (0-1)	0.86 (0.35)	0.85 (0.36)	0.91 (0.29)	0.9 (0.31)	0.85 (0.35)
Poor concentration/psychomotor retardation (0-2)	0.09 (0.3)	0.09 (0.31)	0.13 (0.37)	0.07 (0.27)	0.09 (0.3)
Loss of appetite and body weight (0-1)	0.31 (0.46)	0.31 (0.46)	0.5 (0.5)	0.31 (0.46)	0.3 (0.46)
Increased appetite and body weight (0-1)	0.11 (0.31)	0.14 (0.35)	0.13 (0.33)	0.14 (0.35)	0.1 (0.3)
Insomnia (0-1)	0.24 (0.43)	0.21 (0.41)	0.43 (0.5)	0.23 (0.42)	0.24 (0.43)
Loss of energy/fatigue (0-1)	0.38 (0.48)	0.37 (0.48)	0.57 (0.5)	0.38 (0.49)	0.37 (0.48)
Psychomotor agitation (0-1)	0.19 (0.39)	0.16 (0.37)	0.29 (0.45)	0.21 (0.41)	0.19 (0.39)
Suicidal/homicidal ideation (0-1)	0.28 (0.45)	0.26 (0.44)	0.36 (0.48)	0.27 (0.45)	0.28 (0.45)
Psychotic symptoms (0-3)	0.23 (0.57)	0.22 (0.56)	0.43 (0.75)	0.24 (0.57)	0.21 (0.56)
**History of medical co-morbidities**	No(%)	No.(%)	No.(%)	No.(%)	No.(%)
Congestive heart failure	1857 (10.58)	127 (7.2)	184 (21.05)	134 (8.2)	1412 (10.63)
Chronic pulmonary disease	4319 (24.6)	400 (22.66)	270 (30.89)	373 (22.81)	3276 (24.66)
Diabetes with chronic complications	756 (4.31)	63 (3.57)	64 (7.32)	74 (4.53)	555 (4.18)
Diabetes without chronic complications	2811 (16.01)	254 (14.39)	200 (22.88)	221 (13.52)	2136 (16.08)
Glaucoma	75 (0.43)	6 (0.34)	14 (1.6)	3 (0.18)	52 (0.39)
Hemophilia	16 (0.09)	0 (0)	4 (0.46)	3 (0.18)	9 (0.07)
Hypotension	1169 (6.66)	92 (5.21)	133 (15.22)	110 (6.73)	834 (6.28)
Inflammatory bowel disease	405 (2.31)	37 (2.1)	31 (3.55)	43 (2.63)	294 (2.21)
Lipid disorders	5590 (31.84)	508 (28.78)	372 (42.56)	467 (28.56)	4243 (31.95)
Any malignancy	3824 (21.78)	355 (20.11)	301 (34.44)	374 (22.87)	2794 (21.04)
Any metastatic malignancy	1469 (8.37)	131 (7.42)	127 (14.53)	151 (9.24)	1060 (7.98)
Mild liver disease	2146 (12.22)	214 (12.12)	152 (17.39)	162 (9.91)	1618 (12.18)
Moderate to severe liver disease	211 (1.2)	21 (1.19)	16 (1.83)	17 (1.04)	157 (1.18)
Myocardial infarction	1420 (8.09)	109 (6.18)	133 (15.22)	97 (5.93)	1081 (8.14)
Obesity	3028 (17.25)	363 (20.57)	98 (11.21)	277 (16.94)	2290 (17.24)
Any organ transplantation	320 (1.82)	28 (1.59)	36 (4.12)	23 (1.41)	233 (1.75)
Overweight	452 (2.57)	60 (3.4)	26 (2.97)	38 (2.32)	328 (2.47)
Peptic ulcer	484 (2.76)	38 (2.15)	44 (5.03)	40 (2.45)	362 (2.73)
Peripheral vascular disease	1658 (9.44)	123 (6.97)	168 (19.22)	138 (8.44)	1229 (9.25)
Primary hypertension	6917 (39.4)	584 (33.09)	473 (54.12)	587 (35.9)	5273 (39.7)
Prolonged QTc interval	36 (0.21)	1 (0.06)	4 (0.46)	5 (0.31)	26 (0.2)
Psoriasis	485 (2.76)	38 (2.15)	28 (3.2)	34 (2.08)	385 (2.9)
Chronic renal insufficiency	1170 (6.66)	82 (4.65)	130 (14.87)	83 (5.08)	875 (6.59)
Rheumatic disease	846 (4.82)	52 (2.95)	50 (5.72)	87 (5.32)	657 (4.95)
Secondary hypertension	111 (0.63)	6 (0.34)	9 (1.03)	18 (1.1)	78 (0.59)
Sexual dysfunction	501 (2.85)	75 (4.25)	38 (4.35)	29 (1.77)	359 (2.7)
SLE	210 (1.2)	17 (0.96)	14 (1.6)	27 (1.65)	152 (1.14)
**History of neurological co-morbidities**					
Cerebral vascular disease	1925 (10.96)	126 (7.14)	168 (19.22)	153 (9.36)	1478 (11.13)
Dementia	281 (1.6)	16 (0.91)	48 (5.49)	18 (1.1)	199 (1.5)
Epilepsy	1051 (5.99)	48 (2.72)	76 (8.7)	115 (7.03)	812 (6.11)
Hemiplegia	545 (3.1)	27 (1.53)	45 (5.15)	54 (3.3)	419 (3.15)
Migraine	1650 (9.4)	160 (9.07)	65 (7.44)	194 (11.87)	1231 (9.27)
Multiple sclerosis	196 (1.12)	18 (1.02)	7 (0.8)	24 (1.47)	147 (1.11)
Parkinson’s Disease	170 (0.97)	30 (1.7)	19 (2.17)	14 (0.86)	107 (0.81)
Traumatic brain injury	542 (3.09)	39 (2.21)	42 (4.81)	45 (2.75)	416 (3.13)
**History of psychiatric co-morbidities**					
ADHD	442 (2.52)	82 (4.65)	17 (1.95)	43 (2.63)	300 (2.26)
Alcohol use disorders	1497 (8.53)	167 (9.46)	98 (11.21)	124 (7.58)	1108 (8.34)
Anxiety disorders	6454 (36.76)	581 (32.92)	341 (39.02)	565 (34.56)	4967 (37.4)
Cluster A personality disorder	9 (0.05)	1 (0.06)	2 (0.23)	1 (0.06)	5 (0.04)
Cluster B personality disorder	100 (0.57)	10 (0.57)	4 (0.46)	16 (0.98)	70 (0.53)
Cluster C personality disorder	8 (0.05)	1 (0.06)	0 (0)	1 (0.06)	6 (0.05)
Other personality disorder	101 (0.58)	15 (0.85)	7 (0.8)	8 (0.49)	71 (0.53)
Eating disorders	242 (1.38)	23 (1.3)	12 (1.37)	22 (1.35)	185 (1.39)
PTSD	729 (4.15)	58 (3.29)	39 (4.46)	90 (5.5)	542 (4.08)
Substance use disorders (non-alcohol)	3039 (17.31)	407 (23.06)	194 (22.2)	297 (18.17)	2141 (16.12)
**Total** **N**	17556	1765	874	1635	13282

^a^The value for “depression related symptoms” refers to the number of “concepts” that comprise each symptom (e.g., “depressive mood and anhedonia”) that were recorded by applying NLP to the patient’s clinical note set during the interval from 3 months prior up to the index visit. For example, for “depressive mood and anhedonia,” “anhedonia” and “sadness” are two comprising concepts. The parenthesis after each symptom denotes the number of concepts comprising that symptom – for example, “depressive mood and anhedonia” includes 7 concepts, while “pain” only include one concept. Detailed description of the derivation procedure is provided in Sheu et al. 2022^48^.

Overall, the female/male ratio was 2:1 (66% female), consistent with the known gender ratio of diagnosed depression^45^. The mean (SD) age of the patients was 50.04 (17.59) at the date of index visit. In general, patients started on mirtazapine were older, more likely to be male, and to have had a greater burden of medical illness compared to the other three groups. The bupropion group was slightly younger. There was only a slight variation in depression-related mental symptoms across the four groups (Table 1).

### Model performance metrics

Table 2 reports the point estimates for model performance on the full test set across each setting (the full table with confidence intervals is provided as Supplementary Table 1). All models achieved an area under the receiver operating characteristic curve (AUROC) of at least 70% and an area under the precision-recall curve (AUPRC) of at least 68%, indicating good overall model discrimination. Among the four feed-forward deep learning models, three that included either i) the treatment response likelihood score (a decimal number between 0-1 representing a preliminary evaluation of response probability by a DNN model using clinical notes in a three-year window prior to the index visit) as a predictor or ii) the imputed labels, or iii) both (see Methods for further details) had higher AUROC point estimates than the one without both the treatment response likelihood score and the imputed labels (AUROC for the first three models = 74% and AUROC = 70% for the last model).

**Table 2:** Model performance metrics for antidepressant treatment response prediction.

Model type	Treatment response likelihood score	Imputation^b^	AUROC	AUPRC	Accuracy	F1	NPV	PPV	Sensitivity	Specificity	Threshold^c^
Regularized GLM	yes	yes	0.73	0.71	**0.71**	0.70	0.69	0.72	0.68	0.73	0.50
Regularized GLM	yes	no	0.72	0.71	0.69	0.68	0.68	0.70	0.67	0.71	0.53
Regularized GLM	no	yes	0.73	0.71	**0.71**	0.72	0.73	0.69	0.76	0.65	0.43
Regularized GLM	no	no	0.73	0.70	0.70	**0.74**	**0.77**	0.66	0.83	0.57	0.44
Random forest	yes	yes	0.71	0.70	0.68	0.68	0.68	0.67	0.70	0.66	0.44
Random forest	yes	no	0.73	0.72	0.70	0.67	0.67	0.73	0.63	0.77	0.52
Random forest	no	yes	0.72	0.71	0.69	0.73	0.76	0.65	0.82	0.56	0.37
Random forest	no	no	0.73	0.72	0.70	0.69	0.69	0.70	0.68	0.72	0.47
Gradient boosting	yes	yes	0.73	0.70	0.69	0.65	0.66	0.73	0.59	0.78	0.53
Gradient boosting	yes	no	0.73	0.70	0.69	0.68	0.67	0.70	0.65	0.73	0.53
Gradient boosting	no	yes	0.73	0.71	0.67	0.68	0.69	0.66	0.71	0.64	0.43
Gradient boosting	no	no	0.73	0.71	0.69	0.72	0.76	0.65	**0.82**	0.56	0.38
Transformer + feed-forward DNN	^a^	yes	0.71	0.69	0.68	0.68	0.68	0.69	0.67	0.70	0.42
Transformer + feed-forward DNN	^a^	no	0.72	0.68	0.68	0.67	0.67	0.69	0.66	0.71	0.45
Feed-forward DNN	yes	yes	**0.74**	0.72	0.70	0.70	0.69	0.71	0.68	0.72	0.52
Feed-forward DNN	yes	no	**0.74**	**0.73**	0.70	0.67	0.67	0.73	0.62	0.77	0.56
Feed-forward DNN	no	yes	**0.74**	0.72	0.70	0.71	0.71	0.69	0.73	0.67	0.51
Feed-forward DNN	no	no	0.70	0.70	0.67	0.61	0.63	**0.74**	0.51	**0.82**	0.42

Bold indicates the highest value for the specific metric. Treatment response likelihood score: treatment response likelihood score derived by the NLP prediction model using clinical notes up to 3 years prior to index visit.

^a^Use of vectorized 3-year notes instead of the treatment response likelihood score.

^b^Imputation: inclusion of deep-learning imputed labels in addition to expert-curated labels during training.

^c^Threshold: probability threshold used to derive threshold-dependent metrics; value used was threshold at which model accuracy was maximized.

Given that no model tested was clearly superior across all metrics, and for other reasons discussed later (see Discussion), we selected a “representative model” and report additional model characteristics of this model for the purpose of discussion and illustration. One model that performed well and has several advantages (in terms of extensibility and flexibility) is the feed-forward DNN model that included both the treatment response likelihood score and imputed labels (see Methods). In addition, this model incorporates the broadest range of data among the four feed-forward DNN models and is less computationally intensive than the Transformer + feed-forward DNN model. For this model, AUPRC = 72%, positive predictive value (PPV) = 71%, negative predictive value (NPV) = 69%, sensitivity = 68%, specificity = 72%, F1 score (the harmonic mean of sensitivity and PPV) = 70%, and accuracy = 70%, based on a threshold that maximizes prediction accuracy (See Table 2). Brier score was 0.21. A calibration plot for the model is provided in Supplementary Fig. 1. ROC plots for all models are provided in Supplementary Figs. 2–6.

To contextualize the potential value of the model, we compared the overall prevalence of antidepressant response in the test set data (i.e. the base rate of response independent of applying the model) to the model-predicted response rate. We can think of the overall model-agnostic prevalence as the prior probability of response for these patients based on current practice. We can then compare this to the PPVs obtained with each model in the test data (as a kind of counterfactual response rate had the model been available). For example, the PPV observed with our representative model was 71%, which is statistically significantly higher than the base rate of response (50%) by a two-tailed test for two binomial proportions (z = 7.44, *p* < 0.00001, 95% CI of the difference in the two proportions = (0.16, 0.26)).

**Fig. 2:**
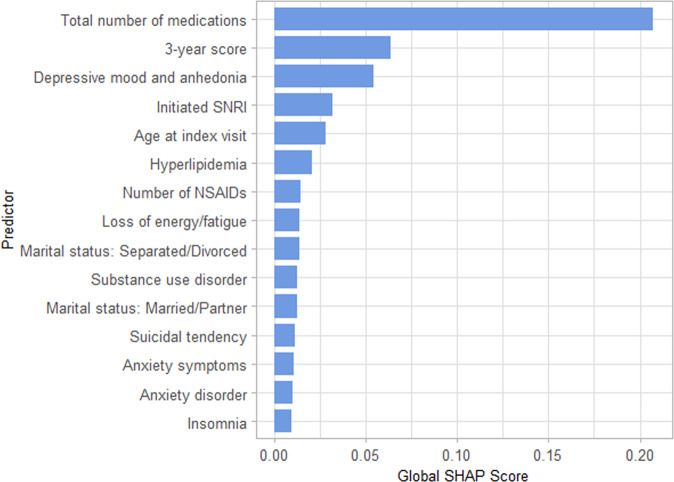
Top 15 features by global SHAP score. Global SHAP scores were calculated by averaging the absolute values of local (i.e., individual) level SHAP scores, and represent overall contribution of the predictors to the model.

Supplementary Table 2 shows performance metrics for the representative model after patient stratification by age, number of co-morbidities, counts of depression-related symptoms, and antidepressant class initiated. Performance of the representative model was generally consistent for all model metrics across most strata. Figure 2 shows the most important predictors overall for the representative model by global sHapley Additive explanation (SHAP) values^46^ (i.e., mean of absolute local SHAP values. Supplementary Fig. 8 shows mean local SHAP values of these important predictors to provide additional information on directionality of feature effects). The top three predictive variables by global SHAP values were the number of co-occurring medications, the treatment response likelihood score, and number of references to depressed mood and anhedonia in clinical notes. The appearance of SNRI initiation among the top 10 predictors confirms that choice of specific antidepressant class is an important contributor to likelihood of responding to an antidepressant.

### Visualization of clinical utility

**Fig. 3:**
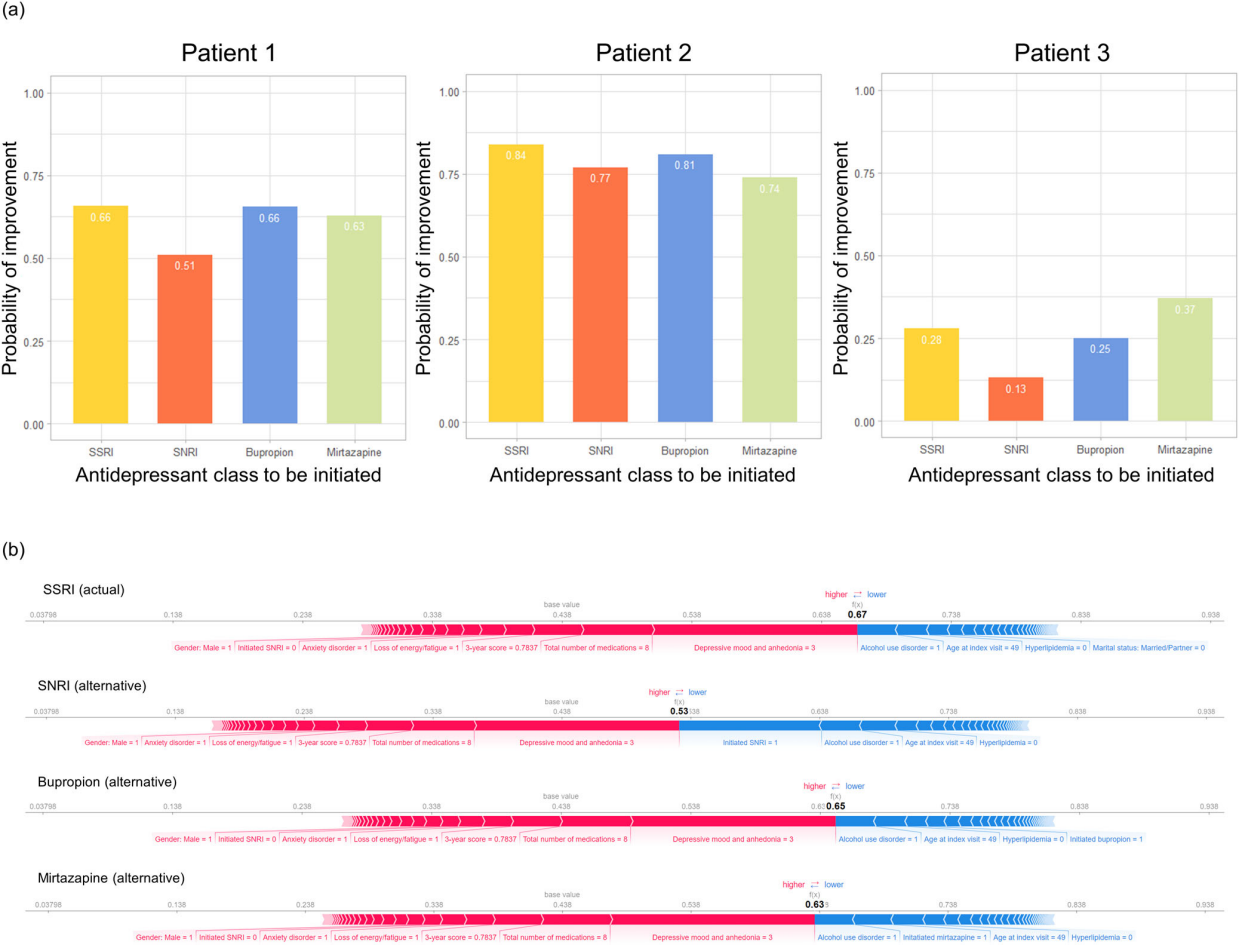
Illustration of differential treatment response predictions and local predictor importance for different treatment scenarios. **a** Predicted response for actual and alternative treatments for three patients randomly drawn from strata that were modeled to be moderately likely (Patient 1), highly likely (Patient 2), and less likely (Patient 3) to respond, respectively, to the actual antidepressant class prescribed. Results are shown for the representative model (feed-forward DNN). **b** “Force plots”^47^ illustrating local predictor importance (by SHAP score) for each of the treatment scenarios for Patient 1 from **a**. The patient was a 49-year-old male with co-morbid alcohol used disorder, depressed mood/anhedonia, anxiety symptoms, fatigue/loss of energy, and 8 co-occurring medications prescribed in the past 3 months, who was started on an SSRI. The directions and strengths of each predictor are shown in directed bars in either blue or red. Only predictors with strengths greater than a threshold level are captioned. The bars denote predictors that decrease (blue) or increase (red) the likelihood of response from base value (i.e., sample mean response probability). Longer bars indicate stronger contributions to predicted response.

To illustrate the kind of information the models can provide, Fig. 3 shows actual and alternative test set predictions and local SHAP predictor importance (i.e., predictor importance for a given patient) for illustrative patients using the representative model (feed-forward DNN). Figure 3a demonstrates predicted response for each antidepressant for three patients drawn from strata that are modeled to be moderately likely (Patient 1), highly likely (Patient 2), and less likely (Patient 3) to respond (see Methods), respectively, to the actual antidepressant class prescribed. Overall, there is substantial variation in predicted treatment response among these patients to any of the four antidepressant classes (e.g., ~80 for Patient 2 and ~0.25 for Patient 3). Within an individual patient, we see more modest variation in predicted response to alternative antidepressants. For example, Patient 1 had a 66% probability of a positive response to an SSRI (the class that was actually prescribed in this case) but only a 51% probability of response had an SNRI been chosen. Figure 3b shows the corresponding local SHAP scores for each initiation scenario for Patient 1. The visualization (“force plot”^47^) displays the relative importance of each predictor to the response prediction for a given antidepressant class. The bolded values are the SHAP model’s overall response prediction for each antidepressant. (Note: the SHAP estimates differ slightly from the DNN estimates (e.g., 0.67 vs 0.66 for SSRI) because they reflect SHAP as a linear estimator of the DNN model; the relative ranking of predicted responses and the feature importance are the same in both models). For each antidepressant, the visualization shows how the predicted probability of response would change given changes in the values of the predictors. Thus, for example, the patient’s response to an SSRI would be predicted to be higher had they not have a diagnosis of alcohol use disorder.

## Discussion

In this study, we developed an AI-assisted machine learning pipeline for antidepressant treatment response prediction using large-scale, real world healthcare data. We systematically examined 14 machine learning models that differed in complexity. The models generated from the pipeline performed generally well by test set metrics, with AUROCs of at least 0.70 and AUPRCs of 0.68 or higher. As a representative example, a feed-forward DNN achieved AUROC of 0.74, and PPV of 0.71 at a threshold that maximized model accuracy. These results show that through appropriate modeling, readily available clinical data (even without formal psychiatric assessment) may provide a basis for the future development of clinical decision support for antidepressant treatment selection. Moreover, we demonstrated how the pipeline could be applied in a clinical context by modeling outcomes for the same patient under each alternative antidepressant treatment scenarios. Using SHAP, we also illustrate the model’s interpretability by displaying the relative importance of predictors to the overall model and to an individual patient’s response to alternative prescription choices. No model was clearly superior to all others based on the full range of performance metrics in the current study. In practice, users might favor some models over others based on other considerations including model interpretability, computational complexity, extensibility (i.e., capability of incorporating additional data modalities), flexibility, or transportability.

A number of prior studies have explored the potential for antidepressant response prediction using a variety of data sources, though all have had limitations. A meta-analysis of 20 studies using some type of machine learning algorithm found none based on large-scale healthcare data and none with a sample size approaching the current study^31^. In addition, in many cases, model performance metrics were either missing or not fully reported. Most studies of antidepressant prediction algorithms have relied on a limited range of predictors, derived largely from demographic variables and symptom scales or have been based on biomarkers (neuroimaging, EEG, or genomics) that are not widely available in clinical practice^30^.

In the feed-forward DNN model we used for illustration, the top 15 features by mean absolute SHAP scores (Fig. 2) include indicators of illness burden as well as specific depression-related symptoms. The top feature was the count of medication prescriptions in the previous 90 days, a possible indicator of psychiatric and medical comorbidity burden. The second-most important feature, the three-year response likelihood score, is a transformation of a patient’s medical history over the prior three years as captured in clinical notes. Several other high-ranking features reflect a range of core depressive symptoms (depressive mood, anhedonia, loss of energy, suicidal tendencies, insomnia) or commonly comorbid conditions that complicate treatment of depression (substance use disorder, anxiety disorder). Patient-specific profiles of these features are commonly considered by clinicians in selecting among classes of antidepressants; as such, they provide some reassurance that influential features selected by the model are consistent with patient factors presumed to be clinically-relevant.

Our study has a number of strengths. First, we performed a systematic examination of models of varying complexity, evaluating the impact of AI-imputed labels and alternative strategies for feature engineering. We demonstrated that AI-assisted label imputation provides a highly scalable alternative to manual chart curation that can otherwise be rate-limiting for model development. Second, we empirically tested the effect on prediction performance of including novel features, including AI-generated vectorized notes and a “treatment response likelihood score” derived from clinical notes. Our motivation for developing these features was the expectation that efficiently incorporating extensive information from longitudinal narrative notes could be helpful. However, in the current study, neither the addition of the 3-year treatment response likelihood score, nor directly using 3-year vectorized notes as features substantially enhanced performance (although the feed-forward DNN models using the response score did show numerical improvement in AUROC). Third, we controlled for confounding by indication by explicitly incorporating potential treatment selection features that might affect treatment response into feature engineering. This allowed us to address the clinically-relevant question for treatment selection: to which major antidepressant classes is a given patient most likely to have a therapeutic response? Finally, the ability to identify which features are most important for predicted response enhances interpretability and gives clinicians insight into individual patient characteristics that are informing the model output.

Our results should also be interpreted in light of several limitations. First, models were trained on labels derived from clinician-documented information on patients’ antidepressant response and augmented with AI-imputed labels. As in any model, misclassification of outcomes used for labeling can impair model accuracy. In addition, EHRs inevitably have missing data, (e.g., patients may receive some of their care outside of the healthcare system), and the model does not explicitly account for effects of concurrent psychotherapy; such missing data may have reduced the predictive performance of the models. Also, while the inclusion of 20 years of EHR can be strength, secular trends in clinician prescribing or documentation practices over this span may have adverse affected model performance. Moreover, as with in any observational setting, there may be residual confounding. However, our approach should minimize confounding by controlling for a broad range of predictors that might influence both antidepressant selection and treatment outcome.

Of note, we applied two data requirements that may impact the generalizability of the model. First, we required at least one clinical note within the 4–12 week period after initiating an antidepressant, which may limit the model’s performance for patients not seen within that window. In training the model, a substantial proportion (70%) of patients started on an antidepressants lacked a clinical note within the following 4–12 weeks. This window was chosen in part because treatment guidelines suggest that 4–8 weeks are typically needed before concluding that an antidepressant is not effective^3^. Beyond 12 weeks, patients may have been more likely to have additional interventions (e.g., antidepressant augmentation or switching) that could complicate outcome assessment. In addition, long prediction windows (e.g. many months) would be less suited to our goal of enhancing expeditious selection of effective treatments. Second, we included only patients with at least one note within 90 days prior to initiating an antidepressant. This was done to minimize confounding by indication as recent symptom severity is associated with clinician antidepressant choice^48^ and also likely to be an important predictor of the outcome (i.e., antidepressant response). To address this, we used recent clinical status (derived by NLP of clinical notes) proximal to the treatment decision to adjust for propensity to select a given treatment. While the 90-day cut-off for “recent” was of necessity arbitrary, we chose it to balance the goal of assessing symptoms proximal to treatment initiation while not overly restricting the window for including such information.

In sum, we present a novel computational pipeline, based on real-world EHR data, for predicting differential response to the most commonly used classes of antidepressants. The resulting models achieved good accuracy, discrimination, and positive predictive value. The models’ ability to predict and compare responses across antidepressant classes could prove valuable for further efforts aiming to provide clinical decision support for prescribers. The approach we demonstrate here could also be adapted to a wide variety of other clinical applications for optimizing and individualizing treatment selection.

## Methods

### Institutional review board approval

All procedures were approved by the Institutional Review Board of Mass General Brigham (MGB) Healthcare System (Boston, Massachusetts, USA, Protocol 2018P000765), with a waiver of consent for the analysis of electronic health record data.

### Data source

The data for the study were extracted from the Research Patient Data Registry (RPDR)^49^ of the MGB Healthcare System. The RPDR is a centralized clinical data registry that gathers clinical information from the MGB system. The RPDR database includes more than 7 million patients with over 3 billion records seen across seven hospitals, including two major teaching hospitals: Massachusetts General Hospital and Brigham and Women’s Hospital. Clinical data recorded in the RPDR includes detailed patient information, encounter meta-data (e.g., time, location, provider, etc.), demographics, diagnoses, laboratory tests, medications, providers, procedures, radiology tests, reasons for visits, and narrative clinical notes^49^.

### Study population

EHR data spanning January 1990 to August 2018 were obtained for adult patients (age ≥ 18 years) with at least one visit with a diagnostic ICD code for a depressive disorder (defined as ICD-9-CM: 296.20–6, 296.30–6, and 311; ICD-10-CM: F32.0–9, F33.0–9) co-occurring with an antidepressant prescription, and at least one ICD code for non-recurrent depression (ICD-9-CM: 296.20–6 and 311; ICD-10-CM: F32.0–9) any time during their history. The first visit with an antidepressant prescription is defined as the “index visit” for each patient. Patients were excluded if: (1) the antidepressant prescription was initiated by a psychiatrist, as we focused on patients initiated by non-psychiatrists in this study; (2) initiated antidepressants not among the four classes of interest, or initiated more than one antidepressant; (3) no clinical notes or visit details were available in the 90 days prior to the index visit date or within 4–12 weeks after the index visit date; (4) first prescription occurred before 1997, the year during which use of the latest antidepressant category (mirtazapine) began; or (5) the patient had a diagnosis of bipolar disorder, schizophrenia, or schizoaffective disorder at or prior to the index visit as antidepressant treatment is less likely to be initiated by non-psychiatrists and depression in the context of bipolar or psychotic disorders might introduce heterogeneity in treatment response. We utilized data from all patients available that met the above inclusion/exclusion criteria in our EHR database. Details of the stepwise sample selection procedure are shown in Fig. 1.

### Constructing the outcome labels

For each patient, notes within the outcome window (4–12 weeks after the index visit) were concatenated as a “note set.” One of the authors (YHS), a psychiatrist, randomly sampled 3600 note sets and manually labeled them into two categories. One category was evidence of improvement, based on the presence of notes within the time window indicating that the patient’s mood was improving, such as “depression is well controlled” or “mood-wise, the patient felt a lot better.” The second category was no evidence of improvement, based on either notes stating the patient’s mood is not improving or is worsening, no documentation of mood status, or evidence that mood status could not be addressed due to medical status (e.g., consciousness change). In note sets where mood status was discussed more than once, the most recent status was taken for labeling. These labels were then incorporated in a deep learning-based text classification model trained on the full set of clinical notes to impute labels for the remaining patients (*N* = 13,956) as described previously^50,51^. In brief, we trained a deep learning model to “emulate” manual chart review and used it to impute labels for notes that were not manually reviewed. The model is a modified version of one described in Sheu and colleagues^51^ and the accuracy of the model is 79% (based on a test set where 45% of the samples are positive).

### Constructing the predictors

Since there is substantial uncertainty about which factors predict response to antidepressants, we constructed a broad set of possible predictors based on prior literature of antidepressant treatment^1,52,53^ as well as interviews with clinicians to identify demographic and clinical factors thought to influence treatment selection. The predictor set was designed to capture factors that might be correlated with “confounding by indication” so that we could control for such confounding in the analyses. The final predictor set include three components: (1) “Structured predictors (Supplementary Table 3),” which included demographics, history of diagnoses preceding the index visit (as binary indicators) as well as counts of concurrent medication prescriptions, NSAID prescriptions, and depressive symptoms mentioned in clinic notes at the index visit and up to 90 days prior. Because depressive symptoms are not readily captured in structured EHR data, these were extracted from clinical notes through application of a set of NLP rules, as previously described^48^. Briefly, these features were extracted and constructed using a hierarchical approach consisting of the following four levels (from highest to lowest): 1. categories of depression-related symptoms (e.g., “depression and anhedonia”); 2. concepts within these categories; 3. specific terms used to describe these concepts; and 4. lexical derivatives and regular expressions (i.e., strings in the form the computer program reads) of the specific terms. Terms extraction (with the handling of negation) was performed with matching regular expressions on the clinical notes for each patient, which were then organized following the hierarchy defined above. The final features built that were included in the predictors were the number of concepts present per category. We also included the antidepressant class initiated at the index visit (as one-hot encoding variables contrasting SNRI, bupropion or mirtazapine vs. SSRI as the reference class). The selection and processing for the structured predictors is described in more detail in Sheu et al. 2022;^48^ (2) “Treatment response likelihood score,” a decimal number between 0-1 representing a preliminary evaluation of response probability by a DNN model, constructed by processing the three-year note sets (i.e., clinical notes in a three-year window prior to the index visit) through a DNN model (“Longformer”)^50^, which was trained on the manually-curated outcome labels (in the training set, as described below). The score was then used as a feature in each of the models we tested (with the exception of the Transformer + feed-forward DNN model); and (3) “Vectorized clinical notes,” instead of a single score, a numerical vector representing the same set of clinical notes as mentioned in (2).

Among 3600 patients with expert-curated labels, we randomly sampled 300 as a hold-out validation set for model tuning, and 600 as a hold-out test set. All performance results are reported based on the hold-out test set.

### Models for treatment response prediction

**Fig. 4:**
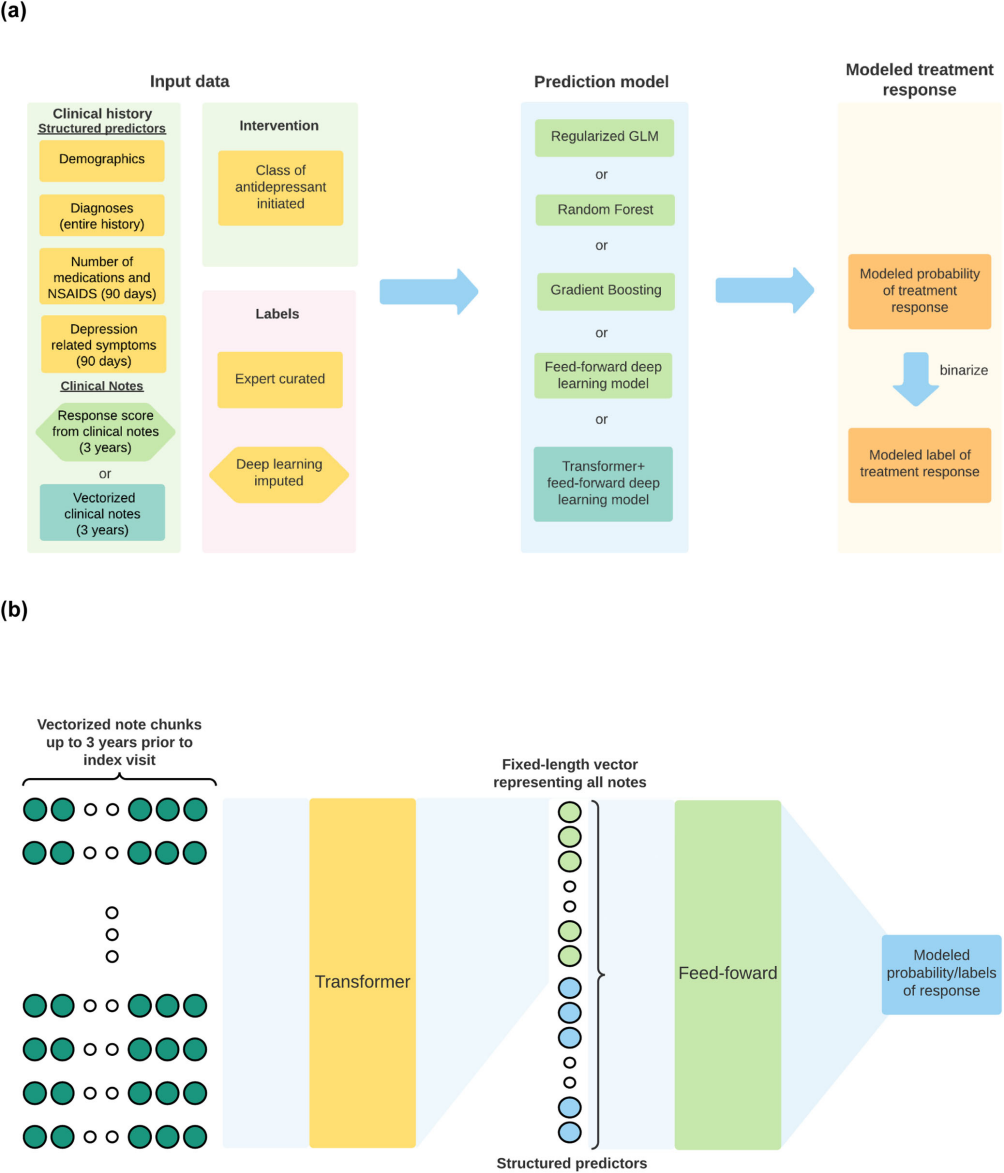
Diagrams for prediction modeling designs. **a** Schematic diagram for modeling antidepressant treatment response (improved vs no evidence for improvement). Model inputs comprised structured and unstructured EHR data regarding demographics and clinical history, choice of antidepressant class, and response outcome labels. Labels were based on chart review by a psychiatrist, plus in some cases by deep learning imputed labels as described in the text. Prediction model outputs modeled probabilities of treatment response, which can be further binarized to a modeled improvement (yes/no) label. Hexagonal boxes indicate data components that were experimentally evaluated for their effect on prediction performance. Yellow boxes indicate data that are used as inputs for every model. Light green and cyan boxes are inputs used only with prediction models shown in matching colors. **b** Schematic diagram for the Transformer + feed-forward DNN model. The model first takes in the vectorized clinical notes through the Transformer, transforms them into a fixed-sized vector, which is concatenated with the other features and then passed through additional feed-forward layers.

We compared five model architectures for prediction (Fig. 4a): (1) regularized generalized linear model (GLM); (2) random forest;^54^ (3) gradient boosting machine (GBM);^55^ (4) feed-forward DNN (with 4 hidden layers); and (5) Transformer^56^ + feed-forward DNN (Fig. 3b). The main difference between models (4) and (5) is that in model (5), we applied an additional “Transformer” component that takes in the structured predictors plus the vectorized notes as inputs (Fig. 4b). For models (1)–(4), we examined performance with and without the 3-year treatment response likelihood score. For all models, we also evaluated whether or not the imputed labels enhanced model performance. All models were trained on a fixed training set, and hyperparameters were tuned to maximize AUROC on the hold-out validation set. For the GLM, we tuned the amount of regularization (lambda), and set alpha at 0.5 (i.e., an elastic net^57^ with both L1 and L2 regularization). For the GBM and random forest models, we tuned on the complexity of the base learners (tree depth) and the total number of trees grown. For deep learning models (model classes (4) and (5)), we tuned on learning rate, dropout probability, and a weight factor that discounts the contribution of patients with imputed labels, based on the confidence of the imputation. We developed the non-deep learning models with R’s h2o package^58^, and the deep learning models with Python, PyTorch^59^, PyTorch-lightning^60^, Huggingface Transformers^61^, and SimpleTransformers^62^. More details regarding model architectures and training processes are provided in Supplementary Methods.

### Assessment of model performance: discrimination and calibration

For all models, we report AUROC, AUPRC, sensitivity, specificity, PPV, NPV, F1 score and accuracy on the test set. Threshold-dependent metrics are reported for the threshold that maximizes accuracy. Although all models performed similarly as shown in Results, we selected a feed-forward DNN as a “representative model” for discussion based on overall consideration of model performance, complexity, flexibility, extensibility, and data representativeness. For this model, we also calculated Brier score, and produced the corresponding calibration plot. To determine if the estimates the model provides are meaningfully different to the current practice, the PPV of the representative model is compared to the base prevalence of response in the test set by a two-tailed Z-test for two proportions and the corresponding confidence interval for the difference in proportions. The base prevalence of response represents the prior probability of response based on clinician judgement.

### Prediction of relative treatment response probability for different antidepressant classes

For any given models tested, four probabilities (one corresponding to each antidepressant class) can be derived for each patient. To illustrate the differential probabilities of antidepressant responses the resulting models can provide at the time when an antidepressant is initiated, we (post-hoc) split the test set by predicted probability of response to a given antidepressant class into high (>70%), medium (40–70%), and low (<40%) using the representative model, based on the actual antidepressant class prescribed. We then sample one patient from each stratum and report the estimated probability of improvement had the patients been treated with each of the other three classes of antidepressants. In this way, we are able to (1) assess whether a patient would response to first-line antidepressants in general; and (2) compare predicted responses to each of the four antidepressant classes for a given patient.

### Global and local predictor importance

For the representative model, we identify which predictors are most important to the overall prediction model (using global SHAP, i.e., mean of absolute local SHAP values)^46^. We also calculate mean SHAP values to assess the overall directionality of the effect of each top feature. Lastly, we illustrate how the model can identify the most important predictors for selecting among the alternative antidepressant classes for a given patient (using the local SHAP metric).

### Reporting summary

Further information on research design is available in the Nature Research Reporting Summary linked to this article.

## Supplementary information

Supplmentary materials

REPORTING SUMMARY

## Data Availability

Protected Health Information restrictions apply to the availability of the clinical data here, which were used under IRB approval for use only in the current study. As a result, this dataset is not publicly available. Qualified researchers affiliated with the Mass General Brigham (MGB) may apply for access to these data the MGB EHR data repository (RPDR) through the MGB Institutional Review Board.

## Code availability

Software packages utilized and cited in this study are available online on their respective websites (h2o for R:^58^ https://cran.r-project.org/web/packages/h2o/index.html; Huggingface Transformers:^61^ https://github.com/huggingface/transformers; SimpleTransformers:^62^ https://simpletransformers.ai; SHAP:^63^ https://github.com/slundberg/shap). Code developed for training of treatment response prediction models can be made available upon reasonable request.
