# Supplementary material for: AI-assisted prediction of differential response to antidepressant classes using electronic health records
Source: NPJ Digit Med. 2023 Apr 26;6:73. doi: 10.1038/s41746-023-00817-8 (PMC10133261; doi:10.1038/s41746-023-00817-8)
Supplement: Supplementary file 1 — Supplmentary materials [file 41746_2023_817_MOESM1_ESM.docx]

**Supplementary On-line Materials**

|  | **Page** |
| --- | --- |
| **Supplementary Table 1** | **3** |
|  |  |
| **Supplementary Table 2** | **6** |
|  |  |
| **Supplementary Table 3** | **7** |
|  |  |
| **Supplementary Figure 1** | **10** |
|  |  |
| **Supplementary Figure 2** | **11** |
|  |  |
| **Supplementary Figure 3** | **12** |
|  |  |
| **Supplementary Figure 4** | **13** |
|  |  |
| **Supplementary Figure 5** | **14** |
|  |  |
| **Supplementary Figure 6** | **15** |
|  |  |
| **Supplementary Figure 7** | **16** |
|  |  |
| **Supplementary Note 1** | **18** |
|  |  |
| **Supplementary Methods** | **21** |
|  |  |
| **Supplementary References** | **24** |

**SUPPLEMENTARY TABLES**

**Supplementary Table 1 | Model performance metrics for antidepressant treatment response prediction**

**Supplementary Table 2 | Model performance for the best model (feed-forward DNN with treatment response likelihood score and imputed labels) stratified by selected patient characteristics**

**Supplementary Table 3 | Variables included in the “structured predictors” set**

**Supplementary Table 1 | Model performance metrics for antidepressant treatment response prediction.** This table provides the same data as Table 2 but also includes 95% confidence intervals around performance metrics.

| **Model type** | | **Treatment response likelihood score** | | **Imputed labels** | **AUROC** | CI 95 | | **AUPRC** | CI 95 | | **Accuracy** | CI 95 | |
| --- | --- | --- | --- | --- | --- | --- | --- | --- | --- | --- | --- | --- | --- |
| Regularized GLM | | | yes | yes | 0.73 | 0.69 | 0.77 | 0.71 | 0.65 | 0.76 | 0.71 | 0.67 | 0.74 |
| Regularized GLM | | | yes | no | 0.72 | 0.68 | 0.76 | 0.71 | 0.65 | 0.76 | 0.69 | 0.66 | 0.73 |
| Regularized GLM | | | no | yes | 0.73 | 0.70 | 0.77 | 0.71 | 0.65 | 0.76 | 0.71 | 0.68 | 0.75 |
| Regularized GLM | | | no | no | 0.73 | 0.69 | 0.77 | 0.70 | 0.64 | 0.75 | 0.70 | 0.67 | 0.74 |
| Random forest | | | yes | yes | 0.71 | 0.67 | 0.75 | 0.70 | 0.64 | 0.74 | 0.68 | 0.65 | 0.72 |
| Random forest | | | yes | no | 0.73 | 0.69 | 0.77 | 0.72 | 0.67 | 0.77 | 0.70 | 0.67 | 0.74 |
| Random forest | | | no | yes | 0.72 | 0.68 | 0.76 | 0.71 | 0.65 | 0.76 | 0.69 | 0.66 | 0.73 |
| Random forest | | | no | no | 0.73 | 0.69 | 0.77 | 0.72 | 0.66 | 0.77 | 0.70 | 0.67 | 0.74 |
| Gradient boosting | | | yes | yes | 0.73 | 0.69 | 0.77 | 0.70 | 0.61 | 0.79 | 0.69 | 0.66 | 0.73 |
| Gradient boosting | | | yes | no | 0.73 | 0.69 | 0.77 | 0.70 | 0.64 | 0.76 | 0.69 | 0.66 | 0.73 |
| Gradient boosting | | | no | yes | 0.73 | 0.68 | 0.76 | 0.71 | 0.65 | 0.76 | 0.67 | 0.65 | 0.72 |
| Gradient boosting | | | no | no | 0.73 | 0.69 | 0.77 | 0.71 | 0.65 | 0.76 | 0.69 | 0.66 | 0.73 |
| Transformer + feed-forward DNN | | | Vectorized notes | yes | 0.71 | 0.66 | 0.75 | 0.69 | 0.62 | 0.74 | 0.68 | 0.65 | 0.72 |
| Transformer + feed-forward DNN | | | Vectorized notes | no | 0.72 | 0.67 | 0.75 | 0.68 | 0.62 | 0.74 | 0.68 | 0.65 | 0.72 |
| Feed-forward DNN | | | yes | yes | 0.74 | 0.70 | 0.78 | 0.72 | 0.66 | 0.76 | 0.70 | 0.68 | 0.74 |
| Feed-forward DNN | yes | | | no | 0.74 | 0.70 | 0.78 | 0.73 | 0.67 | 0.78 | 0.70 | 0.67 | 0.74 |
| Feed-forward DNN | no | | | yes | 0.74 | 0.70 | 0.78 | 0.72 | 0.66 | 0.77 | 0.70 | 0.67 | 0.74 |
| Feed-forward DNN | no | | | no | 0.70 | 0.65 | 0.74 | 0.70 | 0.65 | 0.76 | 0.67 | 0.63 | 0.70 |

**Supplementary Table 1 | Model performance metrics for antidepressant treatment response prediction.** This table provides the same data as Table 2 but also includes 95% confidence intervals around performance metrics (continued).

| **Model type** | **Treatment response likelihood score** | **Imputed labels** | **F1** | CI 95 | | **NPV** | CI 95 | | **PPV** | CI 95 | |
| --- | --- | --- | --- | --- | --- | --- | --- | --- | --- | --- | --- |
| Regularized GLM | yes | yes | 0.70 | 0.64 | 0.74 | 0.69 | 0.64 | 0.75 | 0.72 | 0.67 | 0.78 |
| Regularized GLM | yes | no | 0.68 | 0.63 | 0.73 | 0.68 | 0.63 | 0.74 | 0.70 | 0.65 | 0.76 |
| Regularized GLM | no | yes | 0.72 | 0.67 | 0.76 | 0.73 | 0.68 | 0.79 | 0.69 | 0.65 | 0.74 |
| Regularized GLM | no | no | 0.74 | 0.67 | 0.77 | 0.77 | 0.67 | 0.81 | 0.66 | 0.63 | 0.74 |
| Random forest | yes | yes | 0.68 | 0.62 | 0.73 | 0.68 | 0.63 | 0.76 | 0.67 | 0.63 | 0.74 |
| Random forest | yes | no | 0.67 | 0.63 | 0.74 | 0.67 | 0.64 | 0.75 | 0.73 | 0.66 | 0.78 |
| Random forest | no | yes | 0.73 | 0.64 | 0.76 | 0.76 | 0.66 | 0.80 | 0.65 | 0.63 | 0.74 |
| Random forest | no | no | 0.69 | 0.64 | 0.75 | 0.69 | 0.65 | 0.77 | 0.70 | 0.65 | 0.77 |
| Gradient boosting | yes | yes | 0.65 | 0.60 | 0.74 | 0.66 | 0.63 | 0.77 | 0.73 | 0.64 | 0.78 |
| Gradient boosting | yes | no | 0.68 | 0.62 | 0.74 | 0.67 | 0.63 | 0.75 | 0.70 | 0.65 | 0.77 |
| Gradient boosting | no | yes | 0.68 | 0.59 | 0.75 | 0.69 | 0.62 | 0.79 | 0.66 | 0.63 | 0.77 |
| Gradient boosting | no | no | 0.72 | 0.64 | 0.76 | 0.76 | 0.65 | 0.80 | 0.65 | 0.62 | 0.74 |
| Transformer + feed-forward DNN | Vectorized notes | yes | 0.68 | 0.60 | 0.73 | 0.68 | 0.63 | 0.74 | 0.69 | 0.64 | 0.75 |
| Transformer + feed-forward DNN | Vectorized notes | no | 0.67 | 0.63 | 0.74 | 0.67 | 0.64 | 0.76 | 0.69 | 0.63 | 0.75 |
| Feed-forward DNN | yes | yes | 0.70 | 0.64 | 0.76 | 0.69 | 0.65 | 0.77 | 0.71 | 0.66 | 0.76 |
| Feed-forward DNN | yes | no | 0.67 | 0.63 | 0.75 | 0.67 | 0.64 | 0.78 | 0.73 | 0.65 | 0.78 |
| Feed-forward DNN | no | yes | 0.71 | 0.63 | 0.75 | 0.71 | 0.65 | 0.77 | 0.69 | 0.65 | 0.77 |
| Feed-forward DNN | no | no | 0.61 | 0.55 | 0.69 | 0.63 | 0.59 | 0.69 | 0.74 | 0.64 | 0.81 |

**Supplementary Table 1 | Model performance metrics for antidepressant treatment response prediction.** This table provides the same data as Table 2 but also includes 95% confidence intervals around performance metrics (continued).

| **Model type** | **Treatment response likelihood score** | | **Imputed labels** | **Sensitivity** | | CI 95 | | **Specificity** | | CI 95 | | **Threshold** |  |
| --- | --- | --- | --- | --- | --- | --- | --- | --- | --- | --- | --- | --- | --- |
| Regularized GLM | yes | | yes | 0.68 | | 0.57 | 0.78 | 0.73 | | 0.62 | 0.83 | 0.50 |  |
| Regularized GLM | yes | | no | 0.67 | | 0.57 | 0.80 | 0.71 | | 0.55 | 0.80 | 0.53 |  |
| Regularized GLM | no | | yes | 0.76 | | 0.67 | 0.82 | 0.65 | | 0.59 | 0.75 | 0.43 |  |
| Regularized GLM | no | | no | 0.83 | | 0.65 | 0.86 | 0.57 | | 0.54 | 0.75 | 0.44 |  |
| Random forest | yes | | yes | 0.70 | | 0.56 | 0.85 | 0.66 | | 0.49 | 0.79 | 0.44 |  |
| Random forest | yes | | no | 0.63 | | 0.56 | 0.80 | 0.77 | | 0.60 | 0.82 | 0.52 |  |
| Random forest | no | | yes | 0.82 | | 0.60 | 0.85 | 0.56 | | 0.52 | 0.78 | 0.37 |  |
| Random forest | no | | no | 0.68 | | 0.60 | 0.83 | 0.72 | | 0.55 | 0.80 | 0.47 |  |
| Gradient boosting | yes | | yes | 0.59 | | 0.52 | 0.82 | 0.78 | | 0.55 | 0.85 | 0.53 |  |
| Gradient boosting | yes | | no | 0.65 | | 0.56 | 0.81 | 0.73 | | 0.57 | 0.82 | 0.53 |  |
| Gradient boosting | no | | yes | 0.71 | | 0.50 | 0.88 | 0.64 | | 0.46 | 0.84 | 0.43 |  |
| Gradient boosting | no | | no | 0.82 | | 0.60 | 0.86 | 0.56 | | 0.52 | 0.78 | 0.38 |  |
| Transformer + feed-forward DNN | | Vectorized notes | yes | 0.67 | 0.53 | | 0.76 | 0.70 | 0.59 | | 0.82 | 0.42 | |
| Transformer + feed-forward DNN | | Vectorized notes | no | 0.66 | 0.57 | | 0.81 | 0.71 | 0.55 | | 0.79 | 0.45 | |
| Feed-forward DNN | | yes | yes | 0.68 | 0.57 | | 0.81 | 0.72 | 0.58 | | 0.82 | 0.52 | |
| Feed-forward DNN | | yes | no | 0.62 | 0.55 | | 0.84 | 0.77 | 0.54 | | 0.83 | 0.56 | |
| Feed-forward DNN | | no | yes | 0.73 | 0.56 | | 0.84 | 0.67 | 0.56 | | 0.82 | 0.51 | |
| Feed-forward DNN | | no | no | 0.51 | 0.44 | | 0.74 | 0.82 | 0.58 | | 0.89 | 0.42 | |

**Supplementary Table 2 | Model performance for the representative model (feed-forward DNN with treatment response likelihood score and imputed labels) stratified by selected patient characteristics**. Results indicate that model performance is generally similar across strata, except when the initiated antidepressant was an SNRI.

| **Stratum** | **AUROC** | **AUPRC** | **Accuracy** | **F1** | **NPV** | **PPV** | **Sensitivity** | **Specificity** |
| --- | --- | --- | --- | --- | --- | --- | --- | --- |
| Marginal | 0.74 | 0.72 | 0.70 | 0.69 | 0.69 | 0.71 | 0.68 | 0.72 |
| Age < 65 | 0.74 | 0.73 | 0.71 | 0.72 | 0.70 | 0.72 | 0.72 | 0.70 |
| Age >= 65 | 0.72 | 0.65 | 0.67 | 0.57 | 0.69 | 0.63 | 0.52 | 0.78 |
| Class initiated: bupropion | 0.80 | 0.78 | 0.77 | 0.76 | 0.76 | 0.78 | 0.75 | 0.79 |
| Class initiated: mirtazapine | 0.66 | 0.71 | 0.58 | 0.50 | 0.56 | 0.63 | 0.42 | 0.75 |
| Class initiated: SNRI | 0.58 | 0.37 | 0.70 | 0.36 | 0.79 | 0.38 | 0.33 | 0.83 |
| Class initiated: SSRI | 0.73 | 0.73 | 0.69 | 0.71 | 0.67 | 0.71 | 0.71 | 0.67 |
| Comorbid conditions < 5 | 0.74 | 0.75 | 0.70 | 0.73 | 0.66 | 0.72 | 0.73 | 0.65 |
| Comorbid conditions >= 5 | 0.69 | 0.59 | 0.70 | 0.57 | 0.74 | 0.63 | 0.53 | 0.81 |
| Depression symptoms < 5 | 0.78 | 0.76 | 0.73 | 0.70 | 0.73 | 0.74 | 0.67 | 0.78 |
| Depression symptoms >= 5 | 0.68 | 0.69 | 0.65 | 0.68 | 0.63 | 0.67 | 0.70 | 0.60 |

**Supplementary Table 3 | Variables included in the “structured predictors” set**

| **Antidepressant category first prescribed** | | **History of medical co-morbidities** | |
| --- | --- | --- | --- |
|  | Bupropion |  | Congestive heart failure |
|  | Mirtazapine |  | Chronic pulmonary disease |
|  | SNRI |  | Diabetes with chronic complications |
|  | SSRI |  | Diabetes without chronic complications |
|  |  |  | Glaucoma |
| **Demographics** | |  | Hemophilia |
| *Gender: 2 levels* | |  | Hypotension |
|  | Female |  | Inflammatory bowel disease |
|  | Male |  | Lipid disorders |
| *Race: 6 levels* | |  | Any malignancy |
|  | African American |  | Any metastatic malignancy |
|  | Asian |  | Mild liver disease |
|  | Caucasian |  | Moderate to severe liver disease |
|  | Hispanic |  | Myocardial infarction |
|  | Other |  | Obesity |
|  | Unknown |  | Any organ transplantation |
| *Marital status: 6 levels* | |  | Overweight |
|  | Single |  | Peptic ulcer |
|  | Married/Partner |  | Peripheral vascular disease |
|  | Separated/Divorced |  | Primary hypertension |
|  | Widowed |  | Prolonged QTc interval |
|  | Other |  | Psoriasis |
|  | Unknown |  | Rheumatic disease |
| *Language: 3 levels* | |  | Chronic renal insufficiency |
|  | English |  | Secondary hypertension |
|  | Other |  | Sexual dysfunction |
|  | Unknown |  | SLE |
|  | |  | |
| **Antidepressant and other prescriptions** | | **History of neurological co-morbidities** | |
|  | Age at first antidepressant prescription recorded |  | Cerebral vascular disease |
|  | Number of kinds of co-occurring medications |  | Dementia |
|  | Number of NSAID prescriptions |  | Epilepsy |

**Supplementary Table 3 | List of variables included in the “structured predictors” set (continued)**

| **Depression related symptoms (mean concept counts)** | |  | Hemiplegia |
| --- | --- | --- | --- |
|  | Depressive mood symptoms |  | Migraine |
|  | Poor concentration/psychomotor retardation |  | Multiple sclerosis |
|  | Loss of appetite and body weight |  | Parkinson’s Disease |
|  | Increased appetite and body weight |  | Traumatic brain injury |
|  | Insomnia |  |  |
|  | Loss of energy/fatigue | **History of psychiatric co-morbidities** | |
|  | Psychomotor agitation |  | ADHD |
|  |  |  | Alcohol use disorders |
| **Depression related symptoms (mean concept counts)** | |  | Anxiety disorders |
|  | Suicidal/homicidal ideation |  | Cluster A personality disorder |
|  | Psychotic symptoms |  |  |
|  | Anxiety symptoms | **History of psychiatric co-morbidities** | |
|  | Pain |  | Cluster B personality disorder |
|  |  |  | Cluster C personality disorder |
|  |  |  | Other personality disorder |
|  |  |  | Eating disorders |
|  |  |  | PTSD |
|  |  |  | Substance use disorders (non-alcohol) |

**SUPPLEMENTARY FIGURES**

**Supplementary Figure 1 |** **Calibration plot for the representative model**

**Supplementary Figure 2 |** **ROC curves for GLM models**

**Supplementary Figure 3 |** **ROC curves for random forest models**

**Supplementary Figure 4 |** **ROC curves for gradient boosting machine models**

**Supplementary Figure 5 |** **ROC curves for Transformer + feed-forward DNN models**

**Supplementary Figure 6 |** **ROC curves for feed-forward DNN models**

**Supplementary Figure 7 | Bar Plot of mean SHAP values for the top 15 features**


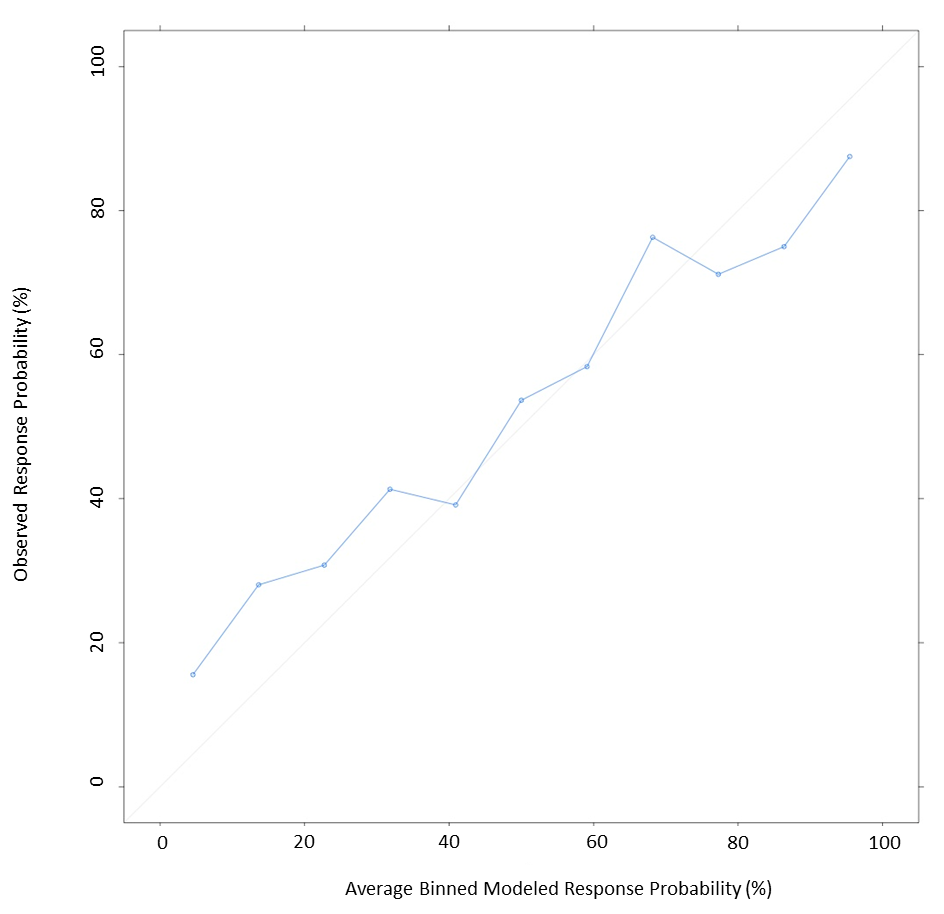


**Supplementary Figure 1 |** **Calibration plot for the representative model.** The X axis represents binned modeled response probability by ordered by ranking, and the Y axis represents observed response probability in the bin. The plot lies roughly on the diagonal, which supports good calibration of the model.

| (a) | (b) |
| --- | --- |
| 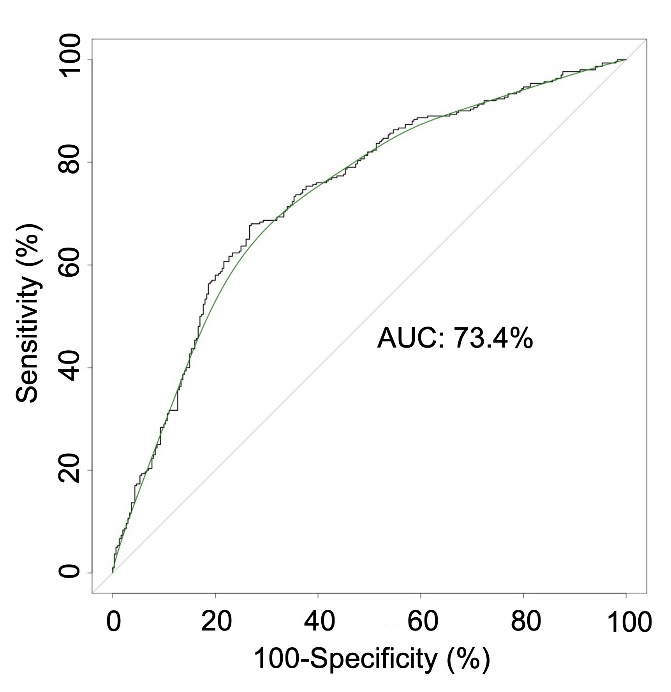 | 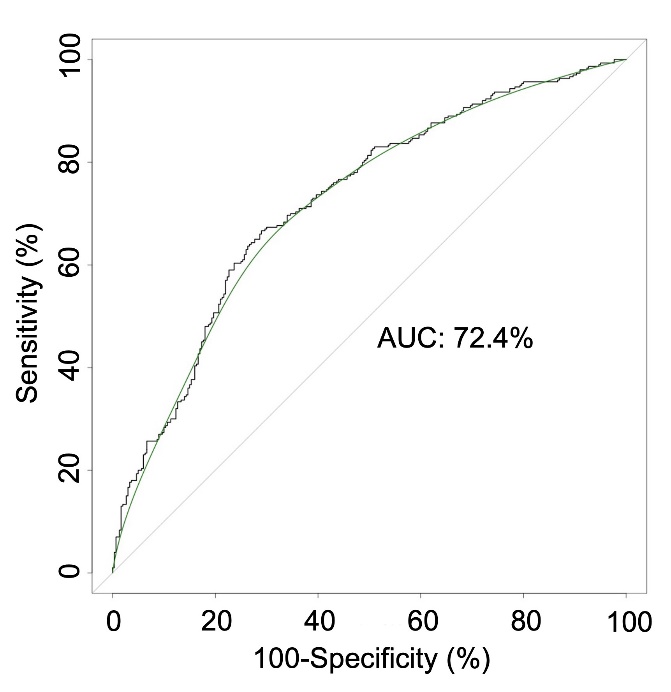 |
| (c) | (d) |
| 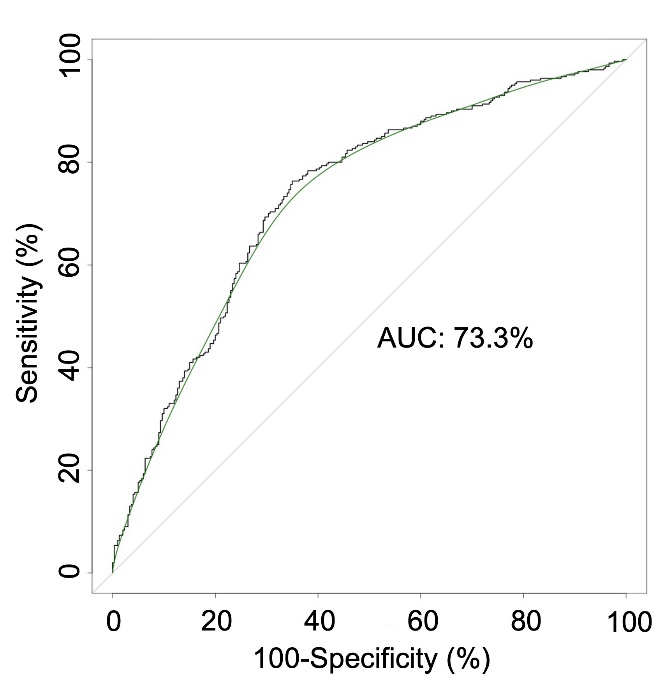 | 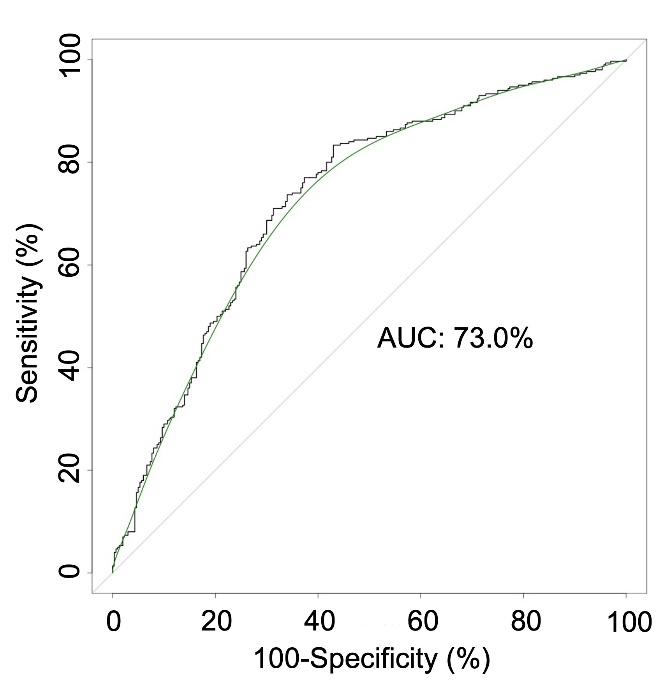 |

**Supplementary Figure 2 |** **ROC curves for GLM models.** (a) Model trained with both treatment response likelihood score and imputed labels; (b) model trained with treatment response likelihood score and without imputed labels; (c) model trained without treatment response likelihood score and with imputed labels; (d) model trained without both treatment response likelihood score and imputed labels.

| (a) | (b) |
| --- | --- |
| 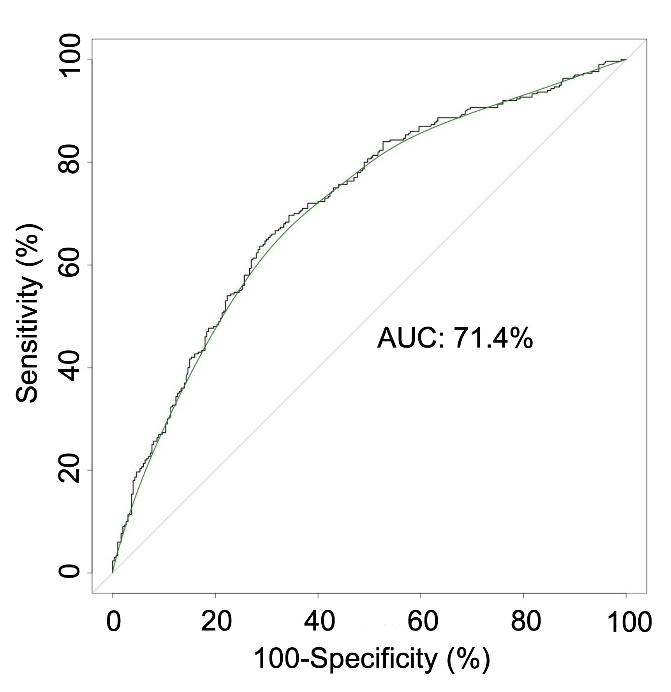 | 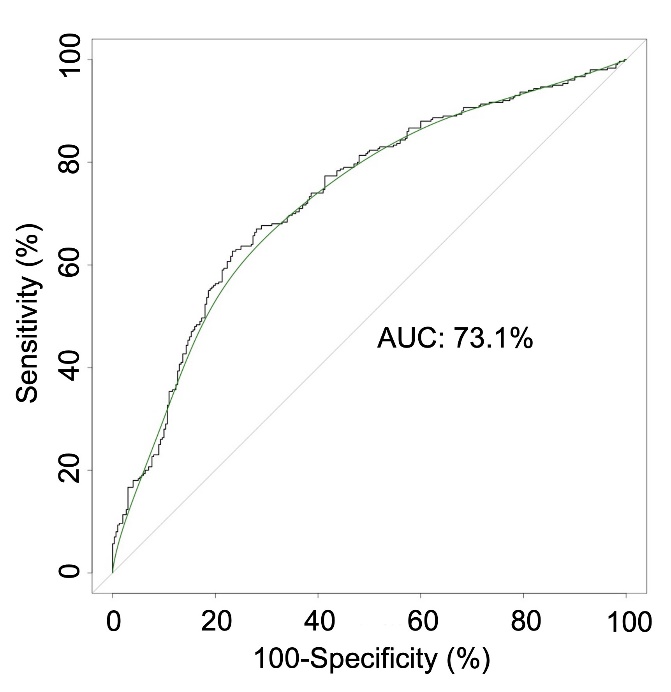 |
| (c) | (d) |
| 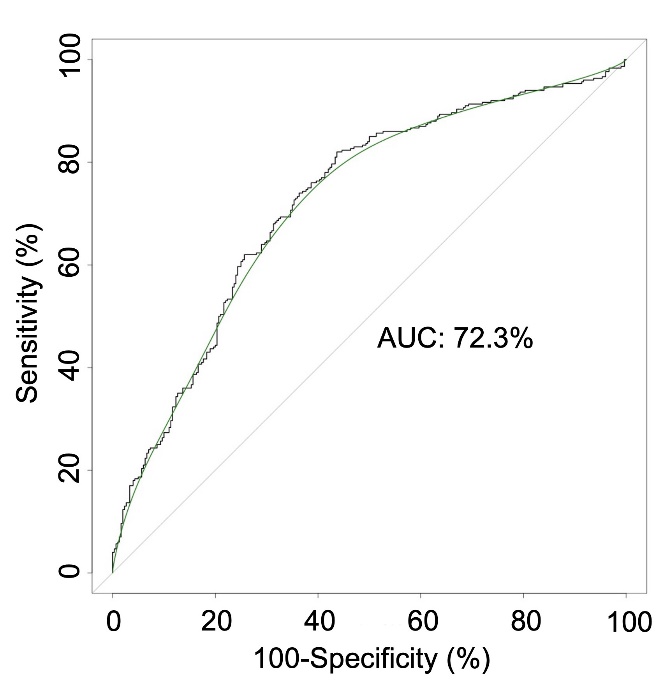 | 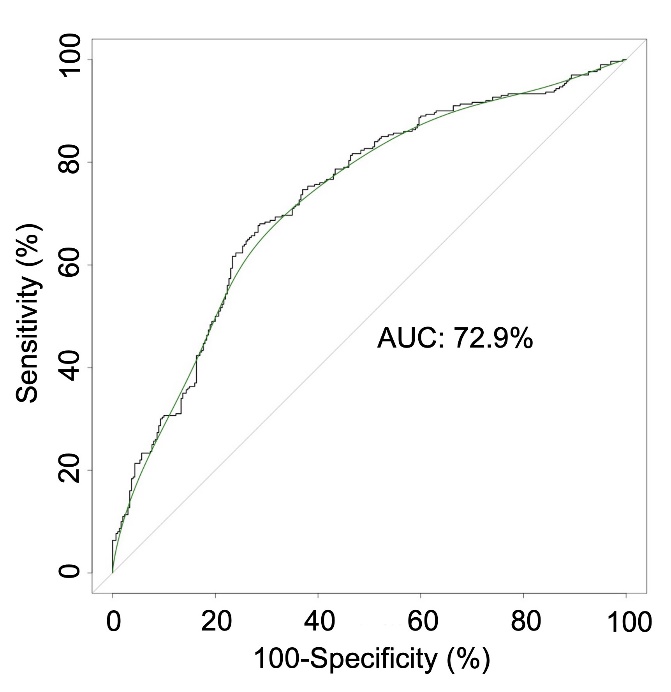 |

**Supplementary Figure 3 |** **ROC curves for random forest models.** (a) Model trained with both treatment response likelihood score and imputed labels; (b) model trained with treatment response likelihood score and without imputed labels; (c) model trained without treatment response likelihood score and with imputed labels; (d) model trained without both treatment response likelihood score and imputed labels.

| (a) | (b) |
| --- | --- |
| 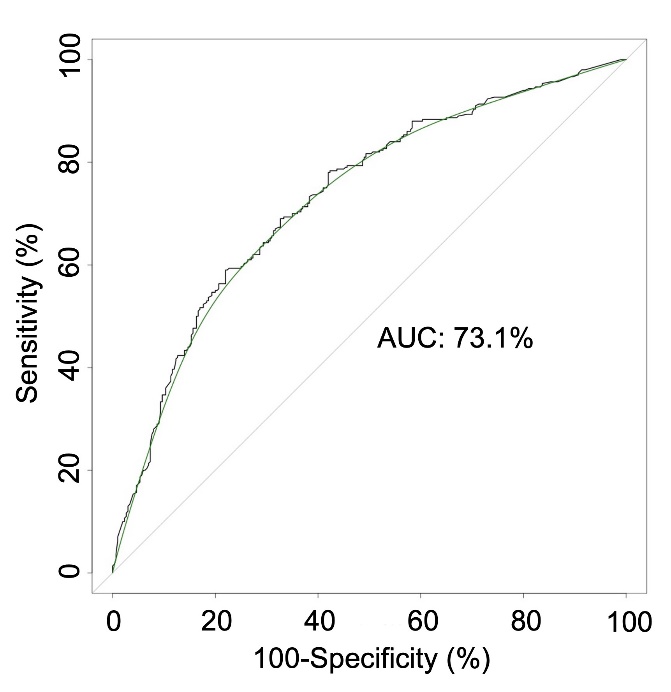 | 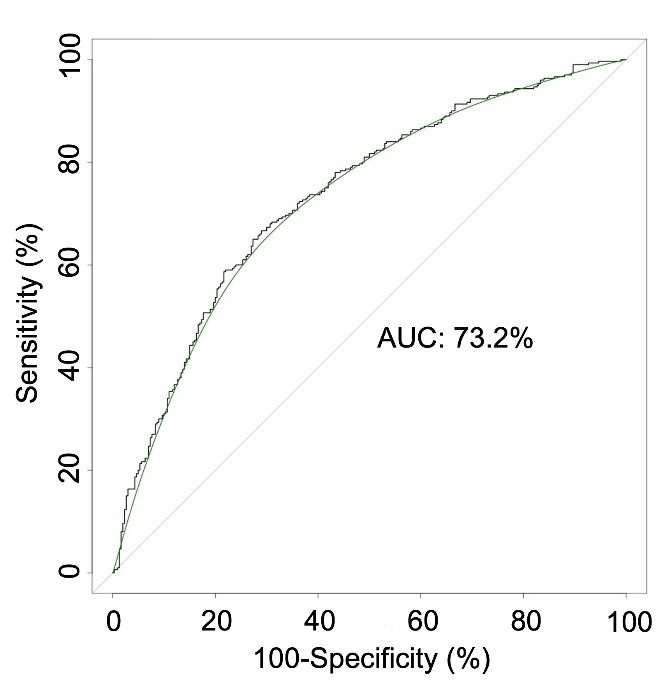 |
| (c) | (d) |
| 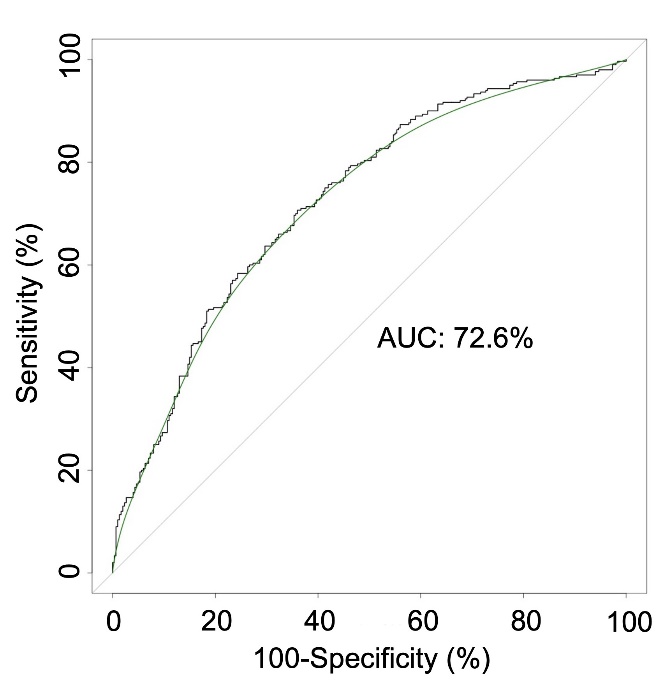 | 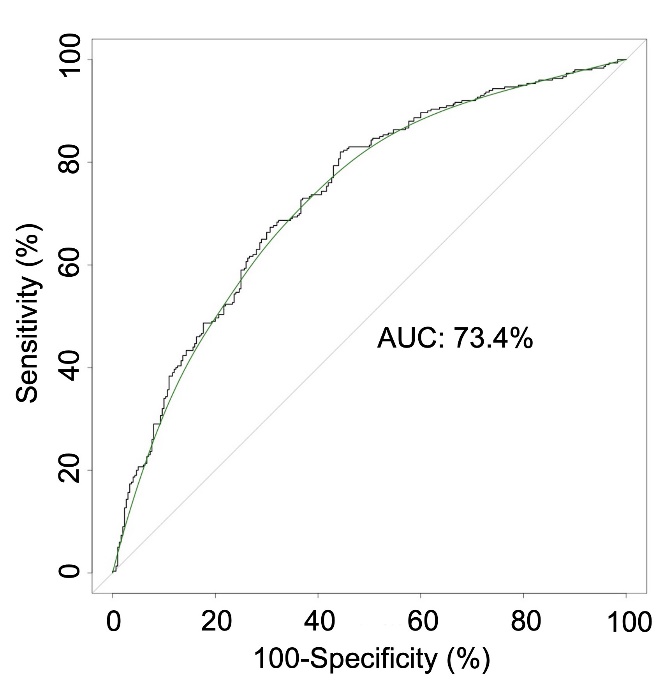 |

**Supplementary Figure 4 |** **ROC curves for gradient boosting machine models.** (a) Model trained with both treatment response likelihood score and imputed labels; (b) model trained with treatment response likelihood score and without imputed labels; (c) model trained without treatment response likelihood score and with imputed labels; (d) model trained without both treatment response likelihood score and imputed labels.

| (a) | (b) |
| --- | --- |
| 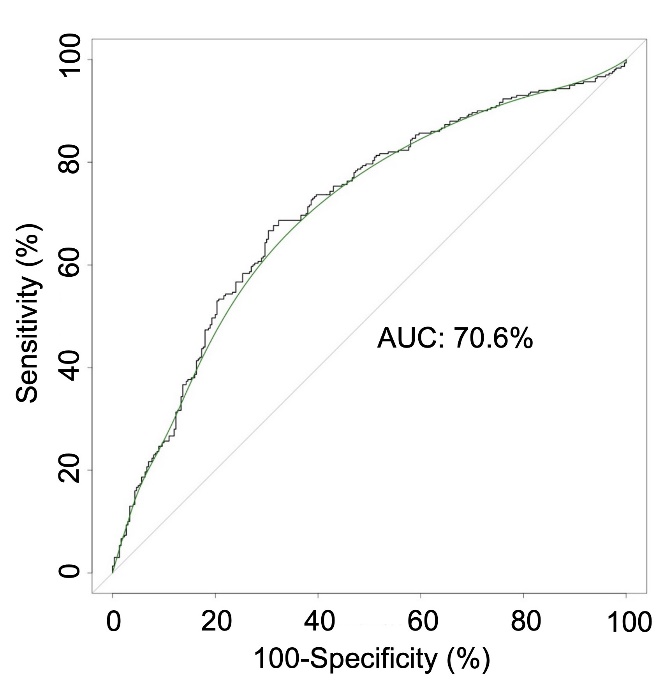 | 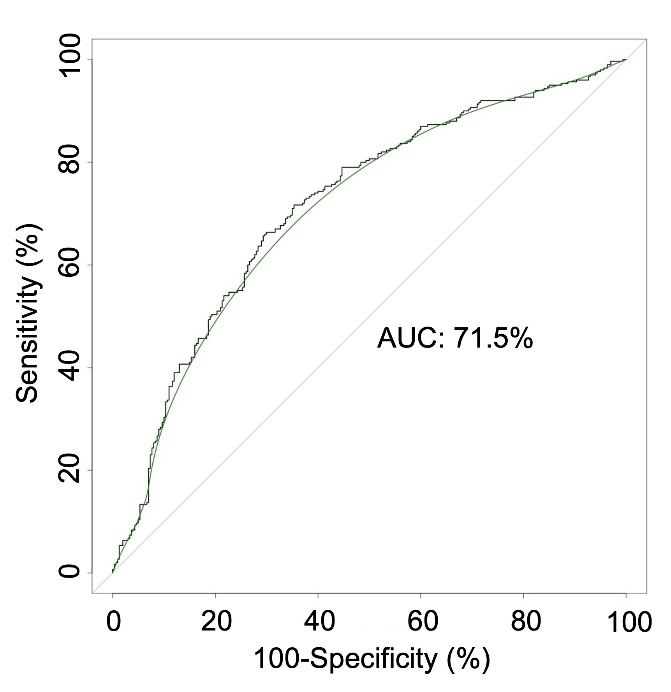 |

**Supplementary Figure 5 |** **ROC curves for Transformer + feed-forward DNN models.** (a) Model trained with imputed labels; (d) model trained without imputed labels

| (a) | (b) |
| --- | --- |
| 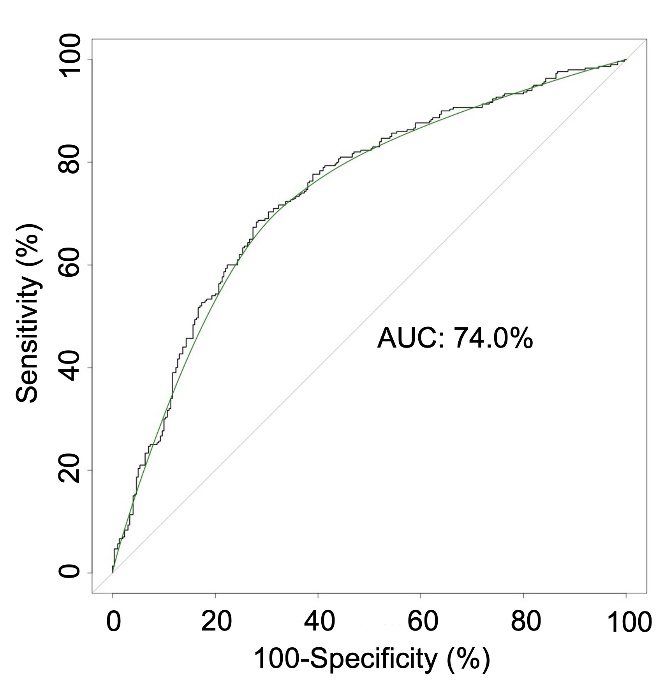 | 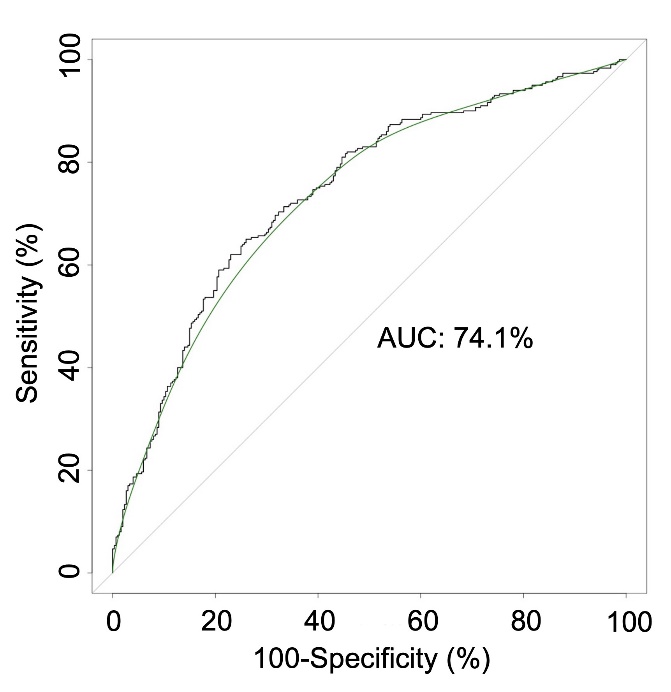 |
| (c) | (d) |
| 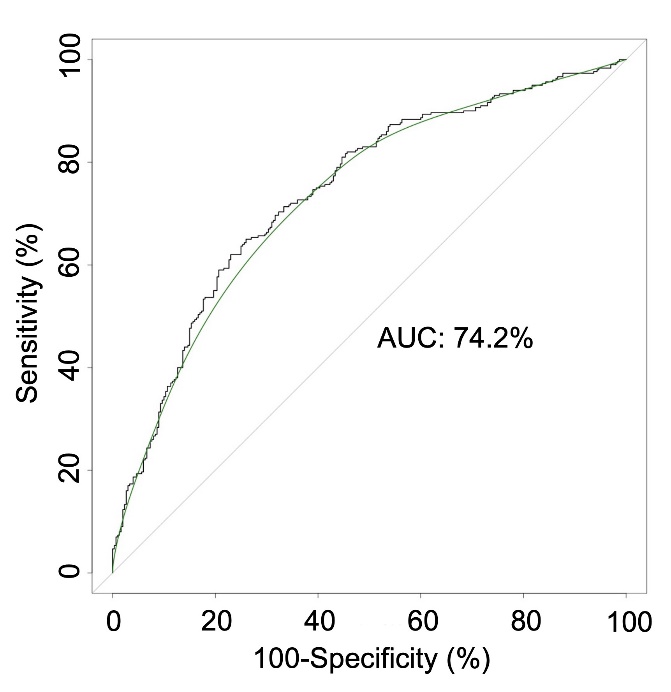 | 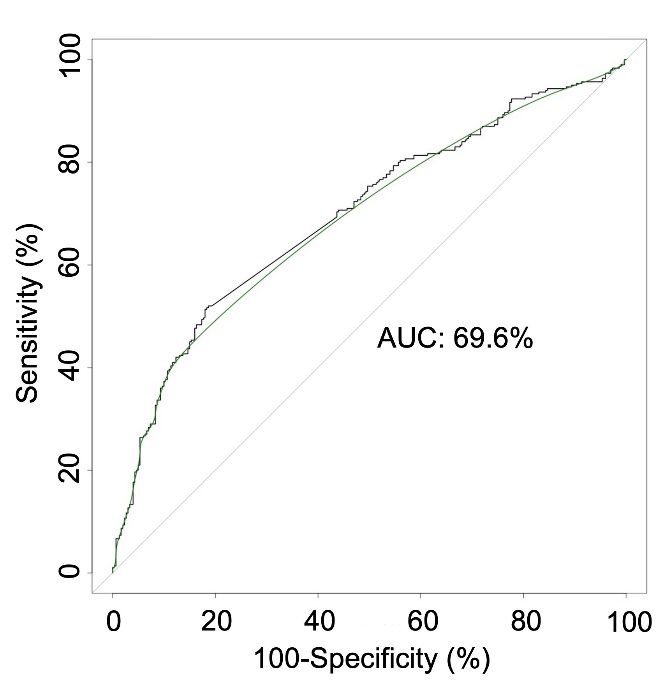 |

**Supplementary Figure 6 |** **ROC curves for feed-forward DNN models.** (a) Model trained with both treatment response likelihood score and imputed labels; (b) model trained with treatment response likelihood score and without imputed labels; (c) model trained without treatment response likelihood score and with imputed labels; (d) model trained without both treatment response likelihood score and imputed labels.


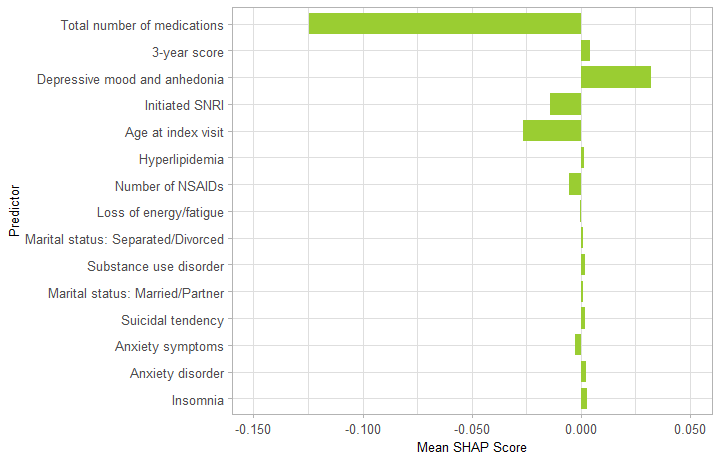


**Supplementary Figure 7 | Bar Plot of mean SHAP scores for the top 15 features.** The values on the x-axis indicate the mean local SHAP score of each of the top 15 features ranked by global SHAP scores (mean of absolute local SHAP scores). A value closer to zero indicates either one or both of the following: (1) positive and negative local SHAP scores were canceled out, or (2) absolute values of local SHAP scores are in general smaller for this feature.

**Supplementary Note**

**Supplementary Note A | About Neural networks (NNs)**

**Supplementary Note A | About Neural networks (NNs)**

A NN[^1^](https://sciwheel.com/work/citation?ids=11146318&pre=&suf=&sa=0&dbf=0) is a type of machine learning model that consists of “artificial neurons,” which are emulations of biological neurons that only output signals when the input signal exceeds a threshold (as in action potentials in their biological counterpart). In DNNs, artificial neurons form “layers.” The neurons within the same layer are not “connected” to one another, i.e., the output of one neuron does not serve as input to another within a layer. A typical neural network is formed by multiple layers that stack up sequentially. Neurons between each layer are connected by numerical weights, which determine the strength of connection between neurons, and are learned during model training. The simplest form of a NN, the feed-forward NN, has every neuron in one layer connected to every neuron in the preceding and subsequent layers. For every NN, the first layer of the network is the “input” layer, the final layer the “output” layer, and layers in between are called the “hidden” layers. NNs with at least one hidden layer are called “deep,” hence Deep Neural Networks (DNNs), or “deep learning.” DNNs are the most common form of contemporary artificial intelligence.

Aside from using artificial neurons as units and the layer-wise structure, the compositions of NNs are flexible, and can be set up in nearly infinite configurations. Therefore, unlike most machine learning approaches where structures are largely fixed, a NN can be tailored for specific purposes and continue to be refined. Architectures that work best for NLP can be very different from those used for imaging data, and the state-of-the-art for each application domain continues to improve over time.

In NLP, the application of DNNs started with recurrent neural networks (RNNs), which offer a natural structure to model sequential data by looking at the input sequence one element at a time, and allowing the information of the hidden layers of one input position to serve as the input for the hidden layers of the next position. RNNs, in conjunction with earlier word embedding approaches such as Word2Vec,[^2^](https://sciwheel.com/work/citation?ids=11146147&pre=&suf=&sa=0&dbf=0) had been the state-of-the-art for DNN-based NLP, until recently when “Transformer,”[^3^](https://sciwheel.com/work/citation?ids=11145859&pre=&suf=&sa=0&dbf=0) a new architecture that uses a blend of feed-forward layers and “self-attention,” was introduced. Self-attention measures similarity between each token in the text, and tokens that are similar and relevant to the task are amplified and emphasized to produce the final model prediction. Models using this approach have shown superior performance, and eventually became the new state-of-the-art for DNN-based NLP.

The next breakthrough after Transformers was the “BERT” model,[^4^](https://sciwheel.com/work/citation?ids=11331484&pre=&suf=&sa=0&dbf=0) which is a large stack of Transformers by design, but instead of having to be trained from scratch, BERT models are contain “pre-trained” model weights that carry information from large text corpora such as Wikipedia, and therefore contain a large amount of syntactical and semantical information before the model is used for downstream tasks. Before downstream applications, the model is further “fined-tuned” – i.e., continues to be trained on the data for the specific task in question, starting from the pre-trained weights. This approach was shown to be very effective and achieved milestone results at the time of its invention.

Nevertheless, because the way self-attention is calculated is memory-demanding, Transformer based models pose a natural limit on the maximum length of input sequences. A series of efforts were taken to overcome that limit, and “Longformer”[^5^](https://sciwheel.com/work/citation?ids=11146095&pre=&suf=&sa=0&dbf=0) is one of the recent approaches that extended the maximum sequence length by 8 times compared to BERT. Longformer can produce either a score or a vector (embedding) that represents the information in the input sequence relevant to the task in question – for example, in our work the score would refer to the probability of response, and the vector would be the embedding that contains information about whether the patient would respond.

**Supplementary Methods**

**Additional Model Training Details for Antidepressant Response Prediction**

**Supplementary Methods | Additional Model Training Details for Antidepressant Response Prediction**

For all model classes mentioned below, except the Transformer[^3^](https://sciwheel.com/work/citation?ids=11145859&pre=&suf=&sa=0&dbf=0) + feed-forward DNN model, each model class was trained with four possible data configurations (i.e., with or without the inclusion of treatment response likelihood score, each with or without the use of imputed labels). Transformer + feed-forward DNN was trained for two data settings (i.e., with or without label imputation). Predictor dimensionality for all model classes, except for the Transformer + feed-forward DNN model, is 75 without the treatment response likelihood score or 76 with the score (i.e., structured features listed in Supplementary Table 3 with categorical variables converted to one-hot encoding and the response likelihood score). The predictor dimensionality for the Transformer + feed-forward DNN model is 75 for the structured predictors. The vectorized clinical notes (described in the main text) were presented as a sequence of embeddings with 1024 dimensions per token. The available labeled data was split into train, validation, and test sets that were mutually exclusive. The treatment response likelihood score and the vectorized notes were derived using the packages Huggingface Transformers[^6^](https://sciwheel.com/work/citation?ids=11146170&pre=&suf=&sa=0&dbf=0) and SimpleTransformers[^7^](https://sciwheel.com/work/citation?ids=11146175&pre=&suf=&sa=0&dbf=0) for Python.

The PyTorch[^8^](https://sciwheel.com/work/citation?ids=11146161&pre=&suf=&sa=0&dbf=0) and PyTorch Lightning[^9^](https://sciwheel.com/work/citation?ids=11146168&pre=&suf=&sa=0&dbf=0) packages for Python was utilized to develop the deep learning prediction models (feed-forward DNN, Transformer + feed-forward DNN). The feed-forward DNN models contain four fully-connected layers, and a dropout layer follows each. The fully connected layers have the following dimensions: 1024, 512, 256, and128, before producing a scalar output.

The Transformer module (defined by nn.TransformerEncoder in PyTorch) for the Transformer + feed-forward DNN model (Figure 4(b)) consists of two Transformer encoder layers with positional encoding and dropout. The Transformer module takes in the sequences of clinical note embeddings as input and has a hidden state of 200 dimensions for each encoder layer. The Transformer model outputs a fixed-length embedding of 300 dimensions. This embedding is then concatenated with the 75-dimension structured predictors before being passed on to three subsequent fully-connected layers, each with 512, 256, and 128 dimensions to derive a scalar output. For both models, all activation functions were ReLUs, and models were trained with BCEWithLogitsLoss and the Adam optimizer.[^10^](https://sciwheel.com/work/citation?ids=12703415&pre=&suf=&sa=0&dbf=0)

For each data configuration of the feed-forward DNN model, models were tuned with the Optuna[^11^](https://sciwheel.com/work/citation?ids=14218110&pre=&suf=&sa=0&dbf=0) package for Python for the following hyperparameters (range in parenthesis): a. *dropout* (0.05, 0.15); b. *learning rate* (1e-6, 6e-3); c. *impute_discount* (0.7, 1.0), which indicates the degree of discount for examples with imputed labels in the loss function to take into account the imperfect accuracy of imputed labels; and d. *impute_cutoff* (0.5, 1.0), a threshold that indicates the level of confidence of an imputed label should exceed for it to be included during training (for the two data configurations without the use of imputed labels, *impute_cutoff* = 1). Hyperparameter combinations of the tuning trials were sampled with a ‘Tree-structured Parzen Estimator (TPE) sampler’ provided by the Optuna package for 1000 trials (i.e., 4000 trials total for each of the four data configurations of the feedforward DNN class). Each trial was trained for 15 epochs. The model state at the epoch with the highest AUROC on the validation set was chosen to represent the specific hyperparameter configuration. The hyperparameter configuration with the highest AUROCs on the validation set was chosen as the final model for each data configuration and was used to report model metrics on the test set.

For the Transformer + feed-forward DNN model, all tuning and model selection procedures are identical to the feed-forward DNN model, except that an additional hyperparameter *nhead* with possible values of (4, 8, 16) was tuned. In addition, models were tuned for 600 trials instead of 1000 (i.e., 1200 trials total for the two data settings).

The R package h2o[^12^](https://sciwheel.com/work/citation?ids=11611641&pre=&suf=&sa=0&dbf=0) was used to train the GLM, random forest, and GBM models. For each of the three model classes, the training set was used to construct an h2o training frame. The validation set was then used to construct an h2o validation frame for the purposes of evaluating hyperparameter candidates. For each model class, the models corresponding to the hyperparameter combinations that yielded the maximum AUC on the validation frame were obtained and utilized to predict the labels on the test set. The grid search strategy for the GLM models was the ‘cartesian’ search strategy, which evaluates every possible combination specified in the grid. The grid search strategy for the GBM and Random Forest models was the ‘RandomDiscrete’ strategy, which performs a random search of the specified grid in combination with an early stopping criterion. The *stopping_rounds* parameter was set to 5, the *stopping tolerance* was set to 1e-4, and the *stopping metric* was set to ‘AUC,’ signifying the training procedure would be early stopped if the AUC did not improve by 0.01% or more for five consecutive rounds of training.

For the GLM models, the hyperparameter grid was comprised of candidate values for *lambda*. This value dictates the strength of regularization via a penalty imposed on the GLM objective function. The *lambda* candidates spanned from 0 to 1 in steps of .001 (1001 total candidates). The *alpha* value specifying the L1 and L2 regularization tradeoff was set at 0.5, indicating an equal distribution between both regularization types. For the GBM models, the hyperparameter grid was comprised of candidate values for *max_depth* (the value that dictates the maximum depth to which a tree can be constructed) and *ntrees*, the total number of trees to build for the model. The *max_depth* candidates spanned from 1 to 30 in steps of 3 (10 total candidates), and *ntrees* spanned from 50 to 500 in steps of 50 (10 total candidates). The *learning rate* was .05, and *learning rate annealing* was .99. *Learning rate annealing* reduced the learning rate by a factor of .99 after each tree was built. The*sample_rate*, which specifies the fraction of data to sample without replacement per individual model, was set at .8. The *col_sample_rate*, which specifies the fraction of features to sample per individual model, was set at .8. The *min_rows*parameter, specifying the minimum number of observations for a leaf in any given tree, was set to 10. For the random forest Models, the hyperparameter grid, search strategy, *col_sample_rate*, and early stopping rules were identical to the GBM models.

**Supplementary References**

[1.    Wang, H. & Raj, B. On the Origin of Deep Learning. *arXiv:1702.07800* (2017).](https://sciwheel.com/work/bibliography/11146318)

[2.    Mikolov, T., Sutskever, I., Chen, K., Corrado, G. S. & Dean, J. Distributed Representations of Words and Phrases and their Compositionality. in *Advances in Neural Information Processing Systems* (eds. Burges, C. J. C., Bottou, L., Welling, M., Ghahramani, Z. & Weinberger, K. Q.) vol. 26 (Curran Associates, Inc., 2013).](https://sciwheel.com/work/bibliography/11146147)

[3.    Vaswani, A. *et al.* Attention is All You Need. in *Proceedings of the 31st International Conference on Neural Information Processing Systems* 6000–6010 (Curran Associates Inc., 2017).](https://sciwheel.com/work/bibliography/11145859)

[4.    Devlin, J., Chang, M.-W., Lee, K. & Toutanova, K. BERT: Pre-training of Deep Bidirectional Transformers for Language Understanding. in *Proceedings of the 2019 Conference of the North American Chapter of the Association for Computational Linguistics: Human Language Technologies, Volume 1 (Long and Short Papers)* 4171–4186 (Association for Computational Linguistics, 2019).](https://sciwheel.com/work/bibliography/11331484)

[5.    Beltagy, I., Peters, M. E. & Cohan, A. Longformer: The Long-Document Transformer. *arXiv:2004.05150* (2020).](https://sciwheel.com/work/bibliography/11146095)

[6.    Wolf, T. *et al.* Transformers: State-of-the-Art Natural Language Processing. in *Proceedings of the 2020 Conference on Empirical Methods in Natural Language Processing: System Demonstrations* 38–45 (Association for Computational Linguistics, 2020). doi:10.18653/v1/2020.emnlp-demos.6.](https://sciwheel.com/work/bibliography/11146170)

[7.    Rajapakse, T. C. Simple Transformers. https://github.com/ThilinaRajapakse/simpletransformers (2019).](https://sciwheel.com/work/bibliography/11146175)

[8.    Paszke, A. *et al.* PyTorch: An Imperative Style, High-Performance Deep Learning Library. in *Advances in Neural Information Processing Systems* (eds. Wallach, H. et al.) vol. 32 (Curran Associates, Inc., 2019).](https://sciwheel.com/work/bibliography/11146161)

[9.    Falcon, W. A. PyTorch Lightning. *PyTorch Lightning* https://github.com/PyTorchLightning/pytorch-lightning (2019).](https://sciwheel.com/work/bibliography/11146168)

[10.   Kingma, D. P. & Ba, J. Adam: A Method for Stochastic Optimization. *arXiv* (2014) doi:10.48550/arxiv.1412.6980.](https://sciwheel.com/work/bibliography/12703415)

[11.   Akiba, T., Sano, S., Yanase, T., Ohta, T. & Koyama, M. Optuna: A Next-generation Hyperparameter Optimization Framework. *arXiv* (2019) doi:10.48550/arxiv.1907.10902.](https://sciwheel.com/work/bibliography/14218110)

[12.   H2O.ai. R Interface for H2O. https://github.com/h2oai/h2o-3.](https://sciwheel.com/work/bibliography/11611641)
