# Supplementary material for: AI-assisted prediction of differential response to antidepressant classes using electronic health records
Source: NPJ Digit Med. 2023 Apr 26;6:73. doi: 10.1038/s41746-023-00817-8 (PMC10133261; doi:10.1038/s41746-023-00817-8)
Supplement: Supplementary file 2 — REPORTING SUMMARY [file 41746_2023_817_MOESM2_ESM.pdf]

nature portfolio

Corresponding author(s): Yi-han Sheu

Last updated by author(s): Mar 22, 2023

Reporting Summary

Nature Portfolio wishes to improve the reproducibility of the work that we publish. This form provides structure for consistency and transparency in reporting. For further information on Nature Portfolio policies, see our Editorial Policies and the Editorial Policy Checklist.

Statistics

For all statistical analyses, confirm that the following items are present in the figure legend, table legend, main text, or Methods section.

n/a	Confirmed	
☐	☒	The exact sample size (*n*) for each experimental group/condition, given as a discrete number and unit of measurement
☐	☒	A statement on whether measurements were taken from distinct samples or whether the same sample was measured repeatedly
☐	☒	The statistical test(s) used AND whether they are one- or two-sided<br>*Only common tests should be described solely by name; describe more complex techniques in the Methods section.*
☐	☒	A description of all covariates tested
☐	☒	A description of any assumptions or corrections, such as tests of normality and adjustment for multiple comparisons
☐	☒	A full description of the statistical parameters including central tendency (e.g. means) or other basic estimates (e.g. regression coefficient) AND variation (e.g. standard deviation) or associated estimates of uncertainty (e.g. confidence intervals)
☐	☒	For null hypothesis testing, the test statistic (e.g. *F*, *t*, *r*) with confidence intervals, effect sizes, degrees of freedom and *P* value noted<br>*Give P values as exact values whenever suitable.*
☒	☐	For Bayesian analysis, information on the choice of priors and Markov chain Monte Carlo settings
☐	☒	For hierarchical and complex designs, identification of the appropriate level for tests and full reporting of outcomes
☐	☒	Estimates of effect sizes (e.g. Cohen's *d*, Pearson's *r*), indicating how they were calculated

Our web collection on statistics for biologists contains articles on many of the points above.

Software and code

Policy information about availability of computer code

Data collection	Electronic Health Records data was requested through data warehouse query. Further data cleaning and pre-processing were performed using R (version 4.0.2) and Python (version 3.7.6)
Data analysis	Software packages utilized and cited in this study are available online on their respective websites (h2o for R 3.36.1.2: https://cran.r-project.org/web/packages/h2o/index.html; Huggingface Transformers 4.2.2: https://github.com/huggingface/transformers; SimpleTransformers 0.46.6: https://simpletransformers.ai/; SHAP 0.39.0: https://github.com/slundberg/shap, Optuna 2.0: https://github.com/optuna/optuna). Non-deep learning prediction models for antidepressant treatment response used in this study were trained and tuned using h2o for R 3.36.1.2. Deep learning prediction models for antidepressant response were implemented using Python 3.7.6, PyTorch 1.6.0, PyTorch Lightning 1.1.8, and models were tuned using Optuna 2.0. The codes used for prediction model development mentioned above are available upon reasonable request.

For manuscripts utilizing custom algorithms or software that are central to the research but not yet described in published literature, software must be made available to editors and reviewers. We strongly encourage code deposition in a community repository (e.g. GitHub). See the Nature Portfolio guidelines for submitting code & software for further information.

Data

Policy information about availability of data

All manuscripts must include a data availability statement. This statement should provide the following information, where applicable:

- Accession codes, unique identifiers, or web links for publicly available datasets
- A description of any restrictions on data availability
- For clinical datasets or third party data, please ensure that the statement adheres to our policy

Protected Health Information restrictions apply to the availability of the clinical data here, which were used under IRB approval for use only in the current study. As a result, this dataset is not publicly available. Qualified researchers affiliated with the Mass General Brigham (MGB) may apply for access to these data the MGB EHR data repository (RPDR) through the MGB Institutional Review Board.

Human research participants

Policy information about studies involving human research participants and Sex and Gender in Research.

Reporting on sex and gender	Patient gender is included in the study as provided by the Mass General Brigham (MGB) Research Patient Data Registry (RPDR), the electronic health records (EHR) data warehouse where data used in this study is queried from under IRB approval. It is not specified whether gender was based on self-report or clinician-documented. The female-to-male gender ratio of the patient sample in this study (11582:5974, approximately 2:1) is consistent with the known gender ratio of diagnosed depression. As stated in the "Data" section above, individual-level Protected Health Information used in this study is not publicly available under the IRB protocol and institutional restrictions. Patient gender is included as a feature for the antidepressant response prediction models developed in the study (i.e., predictions made of individual treatment responses provided by the models factor in patient gender). Feature importance analysis for the prediction model did not indicate gender as an important predictive feature.
Population characteristics	Please see the "study design" section below.
Recruitment	Electronic health records data made available through data warehouse query using the inclusion/exclusion criteria (described in the "study design" section below) under IRB approval. The exclusion criteria imposed requirements on data completeness (measurement of outcome and important predictors), which may limit the models' performance for patients not fulfilling those requirements.
Ethics oversight	This study is approved by the Mass General Brigham (Boston, MA) Institutional Review Board (Protocol 2018P000765), with a waiver of consent for the analysis of electronic health record data.

Note that full information on the approval of the study protocol must also be provided in the manuscript.

Field-specific reporting

Please select the one below that is the best fit for your research. If you are not sure, read the appropriate sections before making your selection.

☒ Life sciences ☐ Behavioural & social sciences ☐ Ecological, evolutionary & environmental sciences

For a reference copy of the document with all sections, see nature.com/documents/nr-reporting-summary-flat.pdf

Life sciences study design

All studies must disclose on these points even when the disclosure is negative.

Sample size	Adult patients (age ≥ 18 years) who were in the EHR data from the Mass General Brigham (MGB) Health Care System spanning January 1990 to August 2018, with at least one visit with a diagnostic ICD code for a depressive disorder (defined as ICD-9-CM: 296.20–6, 296.30–6, and 311; ICD-10-CM: F32.0–9, F33.0–9) co-occurring with an antidepressant prescription, and at least one ICD code for non-recurrent depression (ICD-9-CM: 296.20–6 and 311; ICD-10-CM: F32.0–9) any time during their history. All patients fulfilling the above criteria in the EHR data, less who met the exclusion criteria (described below), were included in the analyses. The final data set comprised 17,556 patients. The sample size is sufficiently large, given the number of predictors included in the prediction models.
Data exclusions	Patients were excluded if they meet any one of the following predefined criteria: (1) had antidepressant treatment initiated by a psychiatrist (the study focused on patients initiated antidepressant by non-psychiatrists); (2) initiated antidepressants not among the four classes of interest, or initiated more than one antidepressant (the study focused on new users of the four first-line antidepressant classes. Patients starting on more than one antidepressant are unlikely to be new users); (3) had no clinical notes available in the 90 days prior to or 4-12 weeks after the index visit date (to ensure depression-related symptoms can be extracted and be used for response prediction and confounding control); (4) had a diagnosis code for bipolar disorder, schizoaffective disorder, or schizophrenia at or prior to the index visit (depression in the context of bipolar or psychotic disorders might introduce heterogeneity in treatment response); and (5) first prescription occurred before 1997, the year during which use of the latest antidepressant category (mirtazapine) began (to ensure all four antidepressant

	classes were available to every patient). Details of the stepwise sample selection procedure with number of patients excluded at each step are shown in Figure 1 in the manuscript.
Replication	Prediction models for antidepressant response were developed on training 16,656 patients and performance were first evaluated on a hold-out set of 300 patients across different hyperparameter settings for optimization. Final model performance is then re-evaluated and reported on a hold-out test set of 600 patients.
Randomization	Treatment assignment is naturalistic (i.e., real-world clinical setting which reflects clinician's treatment selection). Confounding by indication was controlled by applying a broad set of possible predictors based on prior literature of antidepressant treatment as well as interviews with clinicians to identify demographic and clinical factors thought to influence treatment selection.
Blinding	The study is based on observational data and blinding is not relevant.

Reporting for specific materials, systems and methods

We require information from authors about some types of materials, experimental systems and methods used in many studies. Here, indicate whether each material, system or method listed is relevant to your study. If you are not sure if a list item applies to your research, read the appropriate section before selecting a response.

Materials & experimental systems

n/a	Involved in the study
☒	☐ Antibodies
☒	☐ Eukaryotic cell lines
☒	☐ Palaeontology and archaeology
☒	☐ Animals and other organisms
☒	☐ Clinical data
☒	☐ Dual use research of concern

Methods

n/a	Involved in the study
☒	☐ ChIP-seq
☒	☐ Flow cytometry
☒	☐ MRI-based neuroimaging
